# Prediction and classification of ncRNAs using structural information

**DOI:** 10.1186/1471-2164-15-127

**Published:** 2014-02-13

**Authors:** Bharat Panwar, Amit Arora, Gajendra PS Raghava

**Affiliations:** 1 Bioinformatics Centre, Institute of Microbial Technology (CSIR), Sector 39A, Chandigarh, India

**Keywords:** ncRNA, SVM, RandomForest, Graph properties, Prediction, RNAcon

## Abstract

**Background:**

Evidence is accumulating that non-coding transcripts, previously thought to be functionally inert, play important roles in various cellular activities. High throughput techniques like next generation sequencing have resulted in the generation of vast amounts of sequence data. It is therefore desirable, not only to discriminate coding and non-coding transcripts, but also to assign the noncoding RNA (ncRNA) transcripts into respective classes (families). Although there are several algorithms available for this task, their classification performance remains a major concern. Acknowledging the crucial role that non-coding transcripts play in cellular processes, it is required to develop algorithms that are able to precisely classify ncRNA transcripts.

**Results:**

In this study, we initially develop prediction tools to discriminate coding or non-coding transcripts and thereafter classify ncRNAs into respective classes. In comparison to the existing methods that employed multiple features, our SVM-based method by using a single feature (tri-nucleotide composition), achieved MCC of 0.98. Knowing that the structure of a ncRNA transcript could provide insights into its biological function, we use graph properties of predicted ncRNA structures to classify the transcripts into 18 different non-coding RNA classes. We developed classification models using a variety of algorithms (BayeNet, NaiveBayes, MultilayerPerceptron, IBk, libSVM, SMO and RandomForest) and observed that model based on RandomForest performed better than other models. As compared to the GraPPLE study, the sensitivity (of 13 classes) and specificity (of 14 classes) was higher. Moreover, the overall sensitivity of 0.43 outperforms the sensitivity of GraPPLE (0.33) whereas the overall MCC measure of 0.40 (in contrast to MCC of 0.29 of GraPPLE) was significantly higher for our method. This clearly demonstrates that our models are more accurate than existing models.

**Conclusions:**

This work conclusively demonstrates that a simple feature, tri-nucleotide composition, is sufficient to discriminate between coding and non-coding RNA sequences. Similarly, graph properties based feature set along with RandomForest algorithm are most suitable to classify different ncRNA classes. We have also developed an online and standalone tool-- *RNAcon* ( http://crdd.osdd.net/raghava/rnacon).

## Background

The assumption, that proteins are the functional resultant of most genetic information, was derived from studies primarily done on bacteria such as *Escherichia coli* whose genomes are dominated by protein coding sequences (80-95%). The perception that organism (functional) complexity is correlated with the number of protein coding genes was undermined, when by means of sequencing experiments it became abundantly clear that the numbers of protein coding genes do not keep up with the functional repertoire of an organism (Eg: ~1000 cell nematode worm *C. elegans* has ~19,000 genes and which are nearly as many as 10^18^-10^20^ cell humans have (~20,000)). On the other hand, the non-coding region of genomes increases with the complexity of organisms. For example, ~5%, 70% and 80% of the genomic regions of bacteria, unicellular eukaryotes and invertebrates respectively are annotated to be non-coding [1,2]. Amazingly, not only is the majority of this non-coding region transcribed, but also these non-coding RNAs (ncRNA) are proving to be biologically functional [3,4]. Many types of ncRNAs are involved in diverse cellular activities such as replication [5], transcription [6], gene expression regulation (miRNAs: [7]), gene silencing [8,9] and chromosome stability [10], RNA modification (snoRNAs: [11]), RNA processing (RNA subunit of RNase P: [12]), RNA stability [13], protein stability [14], translocation (SRP RNA: [15]) and localization [16]. Having roles in developmental processes and being involved in maintaining homeostasis, any perturbation in the abundances and or sequence of these ncRNAs results in disorders like tumorigenesis [17], neurological disorders [18], cardiovascular [19], developmental [20], autoimmune, imprinting [21] and other human diseases and disorders [22].

Unraveling the functional role of this allegedly inert transcription requires the analysis of large amounts of sequence data. Recently, the ENCODE project [2] assigned biochemical functions for 80% of the human genome, much of which is annotated to be non-protein coding. Various other high-throughput sequencing (HTS) projects are producing huge amounts of transcriptomic data [23]. Thus, computational methods are required to analyze these humongous datasets so as to address the goal of predicting potentially functional non-coding regions and their respective function.

While it has been possible to efficiently discriminate coding and non-coding RNA sequences, for example by employing SVM based prediction models (CONC [24] and CPC [25]), further classifying the non-coding transcripts into functional categories remains challenging. Although, various bioinformatics tools are available for the classification of these transcripts-- their prediction performance is not satisfactory.

Knowing that nucleotide base pairing and stacking interactions between different regions provide ncRNA sequences a well-defined structure; these interactions may also further reveal biological functions. Indeed it has been shown that RNA structure is responsible for specific biological function [26]. Minimum free energy (MFE) based approaches [27] and thermo-stability of multiple aligned structures [28] have also been used for the prediction of functional RNA.

The structure of an ncRNA molecule can be represented as a graph. Being a representative of relationships between different nucleotides, a ncRNA graph uses ‘nodes’ to represent the nucleotides and ‘edges’ to represent the interactions (relationships) between the nucleotides. Such a graph based representation leads to defining of different properties that represent the different characteristics of ncRNA molecules. Two levels of relationships can be defined using graph theory based properties: relationships defined on the level of the nucleotides (‘local properties’) and relationships that represent the graph itself (‘global properties’). Graph properties, derived from a graph representation of predicted ncRNA structure, have been previously used as a feature set to classify ncRNA molecules [29]. Childs *et al*[30] developed a web-based tool, GraPPLE that utilized graph properties to classify RNA molecules into Rfam families. When compared to existing methods, GraPPLE was demonstrated to be more robust to sequence divergence between the members of an Rfam family and also exhibited improved prediction accuracy.

The overall performance of various machine-learning algorithms is intrinsically dependent upon many factors. The performance parameters of GraPPLE could be affected by ‘external’ factors such as the accuracy of predicted RNA structure, the choice of classifier and normalization/optimization procedures selected. In order to explore the potential of different classes of machine learning algorithms to learn distinctive features of ncRNA classes, we employed graph properties as input parameters to a variety of machine learning methods. Additional information regarding structures containing pseudoknot interactions was also incorporated into the modeling framework. So as to facilitate comparative analysis with GraPPLE, we used the same training and testing datasets as GraPPLE. To incorporate RNA pseudoknot information IPknot software was used to predict RNA structures, as it was demonstrated to be more accurate when compared with other methods [31]. We implemented this approach into the form of a web-server (as well as a standalone application) called ‘*RNAcon*’.

## Results

In order to discriminate the non-coding sequences from the coding transcripts, we initially developed a methodology for discriminating non-coding RNA from coding RNAs. Secondly, we developed models to classify ncRNAs into different classes.

### Prediction of non-coding RNAs

**Figure 1:**
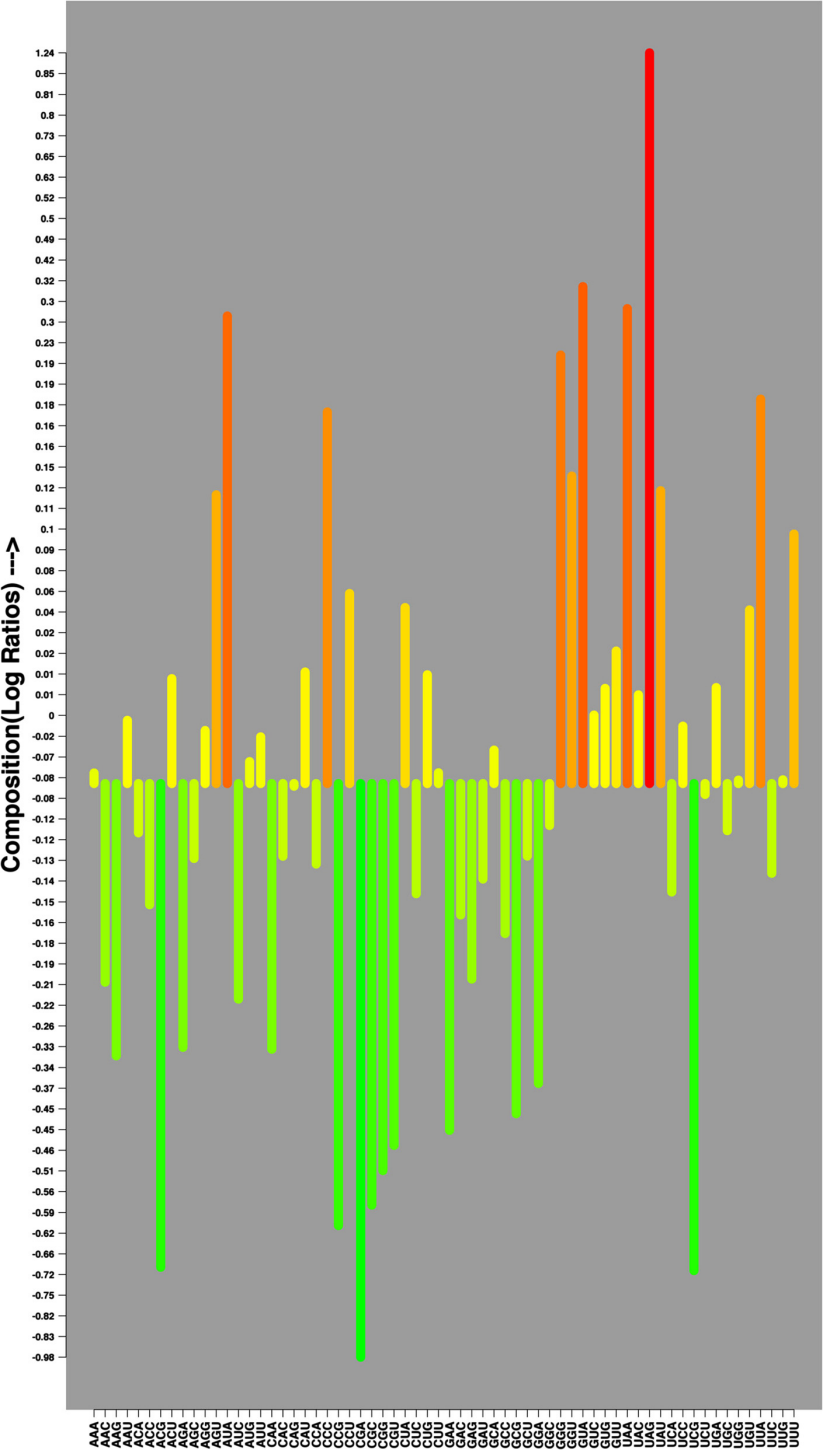
**The comparative average percent tri-nucleotides compositions (TNC) of non-coding and coding RNAs.** The y-axis represents the log2 ratio of non-coding to coding TNC values and the height and color of the bars represents the intensity of the Log2 ratios. The greater TNC of non-coding RNAs can be visualized by the upper (red) shaded bars while the lower panel (green) shows the greater occurrence of a Tri-nucleotide in coding RNA sequences.

We employed composition-based features for discriminating coding and non-coding sequences. Using mono, di, tri, tetra and penta- nucleotides composition as an input feature, machine-learning techniques based models were developed for classification purposes [32]. We analyzed simple mono-nucleotide compositions (MNC) & di-nucleotide compositions (DNC) and observed that Uridine is preferred in noncoding RNA whereas Cytosine and Guanine are more abundant in coding RNA. The comparative analysis of di-nucleotide compositions showed that UA, GU and UU preference in ncRNAs whereas CG, GA, GC, UC, AA and AC are preferred in coding RNAs (Additional file 1: Figure S1). This analysis established the differential nucleotide compositions difference between coding and non-coding RNAs. Thereafter, we used these compositional features for developing SVM-based models. The most efficient model was created by complete optimization of different parameters/kernels and achieved 56.67% sensitivity, 87.13% specificity, 75.08% accuracy and 0.47 MCC using 10-fold cross validation technique. The same procedure was repeated for di-nucleotide compositions (DNC) and achieved 97.13% sensitivity, 94.27% specificity, 95.40% accuracy and 0.91 MCC. The comparative analysis of tri-nucleotide compositions also showed differences between ncRNA and coding RNAs (Figure 1). We achieved 98.90% sensitivity, 99.04% specificity, 98.98% accuracy and 0.98 MCC using 64 input features of tri-nucleotide compositions (TNC). Tetra-nucleotide compositions (TTNC) based approach of 256 input features achieved 99.50% sensitivity, 99.35% specificity, 99.41% accuracy and 0.99 MCC. The 1024 input features of penta-nucleotide compositions (PNC) based approach achieved 98.62% accuracy, 98.55% specificity, 98.58% accuracy and 0.97 MCC. This analysis shows that simple TNC based approach that involves only 64 input features was sufficient to predict ncRNAs with good accuracy (Figure 1). Thereafter, WEKA package [33] was used to select 14 attributes (out of total 64 attributes) of TNC that contributed maximally towards discriminating the coding and non-coding sequences. These tri-nucleotides are ACG, CCG, CGA, CGC, CGG, CGU, CUA, GCG, GGG, GUA, UAA, UAC, UAG and UCG. We applied TNC of these 14 tri-nucleotides for SVM-based prediction and achieved 96.41% sensitivity, 96.49% specificity, 96.46% accuracy and 0.93 MCC.

We also applied a hybrid approach of all five (MNC, DNC, TNC, TTNC and PNC) approaches. Using this, we predicted SVM scores of all five as an input for the SVM based machine learning. On the basis of these five input features, we achieved 99.46% sensitivity, 99.46% specificity, 99.46% accuracy and 0.99 MCC. In the machine learning based predictions, over-optimization is a major problem so it is important to evaluate, models on independent datasets. We used 50% data of both noncoding and coding RNA for training and testing by 10-fold cross validation and predicted remaining 50% independent data on the developed SVM models. Here we achieved 0.46, 0.91, 0.97, 0.98, 0.97 and 0.98 MCC for the MNC, DNC, TNC, TTNC, PNC and Hybrid approaches respectively (Table 1). The performance of SVM based prediction model is threshold-dependent, thus we provided threshold-wise results of all approaches in the Additional file 1: Tables S1, S2, S3, S4, S5 and S6.

**Table 1:** SVM based highest prediction performances (on the basis of MCC) of different composition approaches for the discrimination between non-coding and coding RNAs

**Approach**	**Main dataset**				**Independent dataset**				**CONC dataset**			
	**SN**	**SP**	**ACC**	**MCC**	**SN**	**SP**	**ACC**	**MCC**	**SN**	**SP**	**ACC**	**MCC**
**MNC**	56.67	87.13	75.08	0.47	63.95	81.15	74.34	0.46	50.19	90.48	77.48	0.46
**DNC**	97.13	94.27	95.40	0.91	95.93	95.08	95.42	0.91	81.54	91.15	88.04	0.73
**TNC**	98.90	99.04	98.98	0.98	98.65	98.79	98.74	0.97	86.33	95.39	92.47	0.83
**TTNC**	99.50	99.35	99.41	0.99	99.29	99.05	99.15	0.98	89.51	94.98	93.22	0.85
**PNC**	98.62	98.55	98.58	0.97	98.78	98.29	98.49	0.97	88.43	95.64	93.31	0.85
**Hybrid**	99.46	99.46	99.46	0.99	99.11	99.18	99.15	0.98	89.10	96.29	93.97	0.86

The MNC, DNA, TNC, TTNC and PNC approaches are mono-, di-, tri-, tetra- and penta-nucleotide compositions respectively. Hybrid is a combined approach based prediction of all predicated SVM scores of MNC, DNC, TNC, TTNC and PNC approaches.

### Classification of non-coding RNAs

In the classification of different non-coding RNAs, we used same 20% non-redundant dataset that was earlier used by the GraPPLE method [30]. This dataset incorporated both training and testing datasets for 18 different classes of non-coding RNAs (for detail see Methods section).

#### Composition based approaches

**Figure 2:**
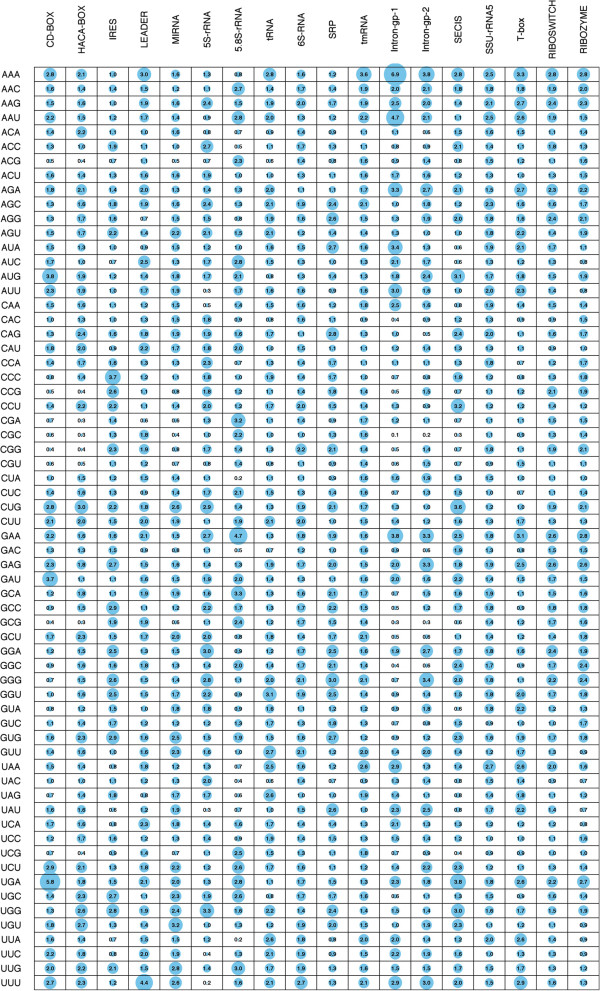
**The comparative average percent tri-nucleotides compositions (TNC) of different non-coding RNA classes for the complete dataset.** The diameter of the bubble is scaled according to the TNC values (the value is also numerically shown inside the bubble).

Knowing that the composition based approaches performed well for the purpose of discriminating coding and non-coding RNAs, we calculated mononucleotide and di-nucleotide composition based differences between the ncRNA classes (Additional file 1: Figure S2 and S3). The tri-nucleotide compositional differences were also different for the whole dataset (Figure 2) and 20% non-redundant dataset (Additional file 1: Figure S4). To check whether these approaches could also classify different ncRNA classes, we used MNC, DNC and TNC as an input feature set. After applying 7 different classifiers (BayeNet, NaiveBayes, MultilayerPerceptron, IBk, libSVM, SMO and RandomForest) it was observed that composition based approaches were unable to classify different ncRNAs. Only BayesNet achieved highest 0.284 sensitivity using tri-nucleotide compositions (Table 2). This suggests that simple composition based input feature set is not able to distinguish between different classes of ncRNAs.

**Table 2:** **Overall sensitivity (Q**^**D**^**) of different classifiers for the classification of 18 ncRNA classes**

**Name of classifier**	**MNC**	**DNC**	**TNC**	IPknot^*^	**IPknot**^**#**^
**BayesNet**	0.073	0.084	0.284	-	0.397
**NaiveBayes**	0.057	0.074	0.058	0.315	0.398
**MultilayerPerceptron**	0.060	0.075	0.128	0.314	0.429
**IBk**	0.057	0.074	0.089	0.314	0.407
**libSVM**	0.060	0.079	0.108	0.214	0.056
**SMO**	0.073	0.081	0.102	0.283	0.422
**RandomForest**	0.055	0.079	0.121	0.400	0.433

^*^IPknot – Normalized (-1.0 to 1.0) graph properties value used for all classifier.

^#^IPknot – Real graph properties value used for all classifiers.

#### Graph properties based approach

**Figure 3:**
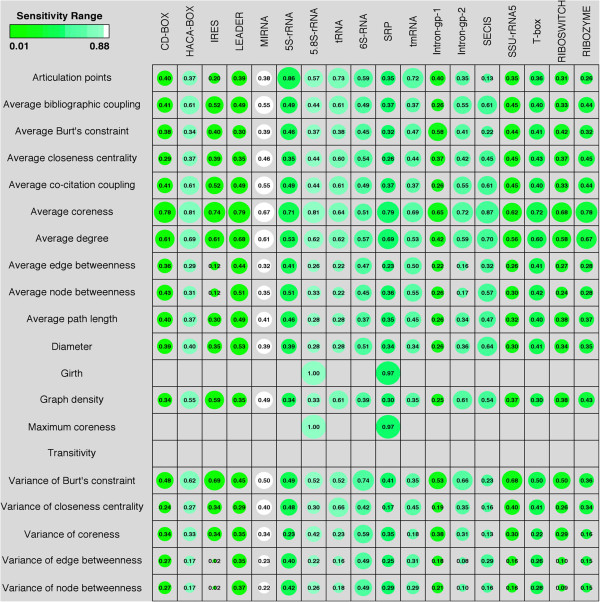
**Relative Graph Properties of different non-coding RNA classes.** The diameter of the bubble is scaled according to the value of the normalized (between 0 to 1) graph properties (the value is also numerically shown inside the bubble). The sensitivity of the prediction has been depicted by the color range (increasing from Green to White) of the bubbles. As can be seen miRNAs have the greatest sensitivity where as LEADER RNAs have the least prediction sensitivity. The blank boxes are where graph property values were predicted to be 0 or null.

As the composition-based approach was not able to classify different classes of ncRNA sequences, an alternative feature set was required that incorporates ncRNA family specific information. It has been shown previously that the structure of ncRNA may provide insights into biological functions and therefore specific ncRNA families [26]. In order to use this ncRNA structural information as a feature set for machine-learning techniques, we firstly predicted the structures of non-coding RNAs belonging to 18 different classes by using IPknot software [31]. The igraph R package was used to calculate the graph properties of all the predicted structures [34]. Total 20 different graph properties were chosen (see detail in Methods). It is evident from Figure 3 (Diameter of data points: Bubble plot) that graph properties contributed differentially towards various ncRNA classes.

As graph properties can represent nucleotide level (local) and structure level (global) parameters, the scale and the range of graph properties metrics vary extensively. So as to provide uniformity in the scale and range of these metrics, we normalized graph properties values into the range of -1.0 to +1.0 before applying 6 different classifiers. The NaiveBayes, MultilayerPerceptron, IBk, libSVM, SMO and RandomForest achieved sensitivity (Q^D^) values of 0.315, 0.314, 0.314, 0.214, 0.283 and 0.400 respectively (Table 2). As the BayesNet classifier failed to run on the normalized values, we tried all these classifiers on the raw, non-normalized, value of graph properties: where BayesNet, NaiveBayes, MultilayerPerceptron, IBk, libSVM, SMO and RandomForest achieved sensitivity values of 0.397, 0.398, 0.429, 0.407, 0.056, 0.422 and 0.433 respectively (Table 2). It must be pointed out that RandomForest (100 tree and 10 seed values) based approach achieved the highest sensitivity of 0.433 and 0.40 MCC (Additional file 1: Figure S5). The MultilayerPerceptron is second highest performing classifier and achieved sensitivity of 0.429 and 0.395 MCC (Additional file 1: Figure S6). SMO classifier based prediction achieved sensitivity of 0.422 and 0.388 MCC (Additional file 1: Figure S7).

Since we used the dataset from the previous study of GraPPLE, where they have already removed the biasness of sequence similarity, length and GC content in order to test the predictive power of graph-properties only [30]. However, we calculated the correlation between the average length of particular ncRNA class and their prediction performance [sensitivity (Q^D^)] from RandomForest model and achieved correlation coefficient values (R) of -0.343 between the average length and sensitivity (Additional file 1: Table S7). It means, length affects the prediction performance and has a negative correlation with the sensitivity.

### Comparisons of *RNAcon* with different existing methods

In order to evaluate the prediction performance of RNAcon (both prediction and classification of noncoding RNAs), we compared RNAcon with different gene-calling programs, CONC, CPC, GraPPLE and Rfam-based covariance models.

#### Comparison with different gene-calling programs

The gene-calling programs detect protein-coding part in the transcripts/cDNAs; therefore, they can also be used to discriminate between coding and noncoding genes. In this study, we used 2670 noncoding RNAs as positive and 5601 coding RNAs as negative datasets-- collectively called CONC dataset. RNAcon achieved 86.25% sensitivity, 90.52% specificity, 89.14% accuracy and 0.76 MCC on this CONC dataset. We evaluate performance of three commonly used gene-calling programs (AUGUSTUS [35], GeneMark.hmm [36] & Glimmer.HMM [37]) on this CONC dataset.

First, we used the standalone version 2.7 of AUGUSTUS on the CONC dataset and found that it predicted genes (protein-coding region) in the 11 (False negatives; 0.41%) noncoding RNAs out of 2670 noncoding RNAs. This means that AUGUSTUS correctly predicts 2659 (True positives) noncoding RNAs as non-coding (99.59% sensitivity). Similarly, it predicted genes in the 1111 (True negatives) coding RNAs out of total 5601 coding RNAs and it failed to predict any gene in the remaining 4490 (False positives) coding RNAs (19.84% specificity). Overall AUGUSTUS achieved 89.14% accuracy and 0.76 MCC. Likewise, GeneMark.hmm (version 2.2b) achieved 90.22% sensitivity, 71.17% specificity, 77.32% accuracy and 0.57 MCC. Comparatively, Glimmer.HMM (version 3.0.3) performed better than other two gene-calling programs and achieved 95.73% sensitivity, 71.68% specificity, 79.45% accuracy and 0.63 MCC. These results showed that RNAcon performed better (0.76 MCC) than other three gene-calling programs (Additional file 1: Table S8).

The prediction of the three selected gene-calling programs is based on the prediction of protein coding genes only. These three algorithms were designed to specifically predict the protein coding genes and ignore the rest of the sequences, treating them as background noise. These gene-calling algorithms perform satisfactorily while predicting non-coding RNAs. In reality these methods are actually ignoring the non-coding "background" by just selecting for the protein coding sequences whereas RNAcon is actually discriminating coding the non-coding genes and not selectively identifying one class from the datasets.

#### Comparison with CONC and CPC

In the discrimination between noncoding and coding RNA, CONC [24] has used various input features (Total 180) such as peptide length, amino acid compositions, secondary structure content, percentage of residues exposed to solvent, sequence compositional entropy, number of homologs in a protein database and alignment entropy. The CONC method was further improved by CPC [25] method using the following six input features: LOG-ODDS score, coverage of the predicted ORF, integrity of predicted ORF, number of hits against protein database, HIT-SCORE and FRAME-SCORE. Using all these complex input features CPC reported 95.77% accuracy. For comparison purposes, we used CPC standalone software [25] to calculate these input features from the same CONC dataset. By developing SVM-based models we achieved maximum accuracy of 94.14%, which is almost similar to five-input features (predicted SVM scores of different compositional features) based hybrid approach of RNAcon (93.97% accuracy) (Table 1). Varied factors such as learning parameters, optimization procedure and SVM version can affect this marginal performance difference. Importantly, RNAcon uses computationally simpler methodology to achieve comparable results. Considering the humongous amounts of sequence data, simple, fast and straightforward methods are requirement of the current times. For example, the RNAcon_predict (standalone version) takes less than 1 minute (0 m58.830 s) to process 30770 sequences (Rfam) using a Mac OS X (version 10.7.5) of 2.5 GHz (Intel Core i5) and 4GB RAM (1333 MHz DDR3) system. In contrast, CPC reported that their method took 3513 minutes of CPU time (Intel Xeon 3.0G and 4GB RAM) for same number (30770) of Rfam sequences. The huge amount of available transcriptomic/NGS data requires RNAcon type of method because it can easily process millions of sequences within reasonably CPU time. The RNAcon_predict processed 100000 sequences each of coding and noncoding RNA in the 6 (5 m51.616 s) and 3 (2 m52.918 s) minutes respectively. Moreover, given the importance of ncRNAs in biology, our primary emphasis was to develop a method for the ncRNA classification.

#### Comparison with GraPPLE

**Figure 4:**
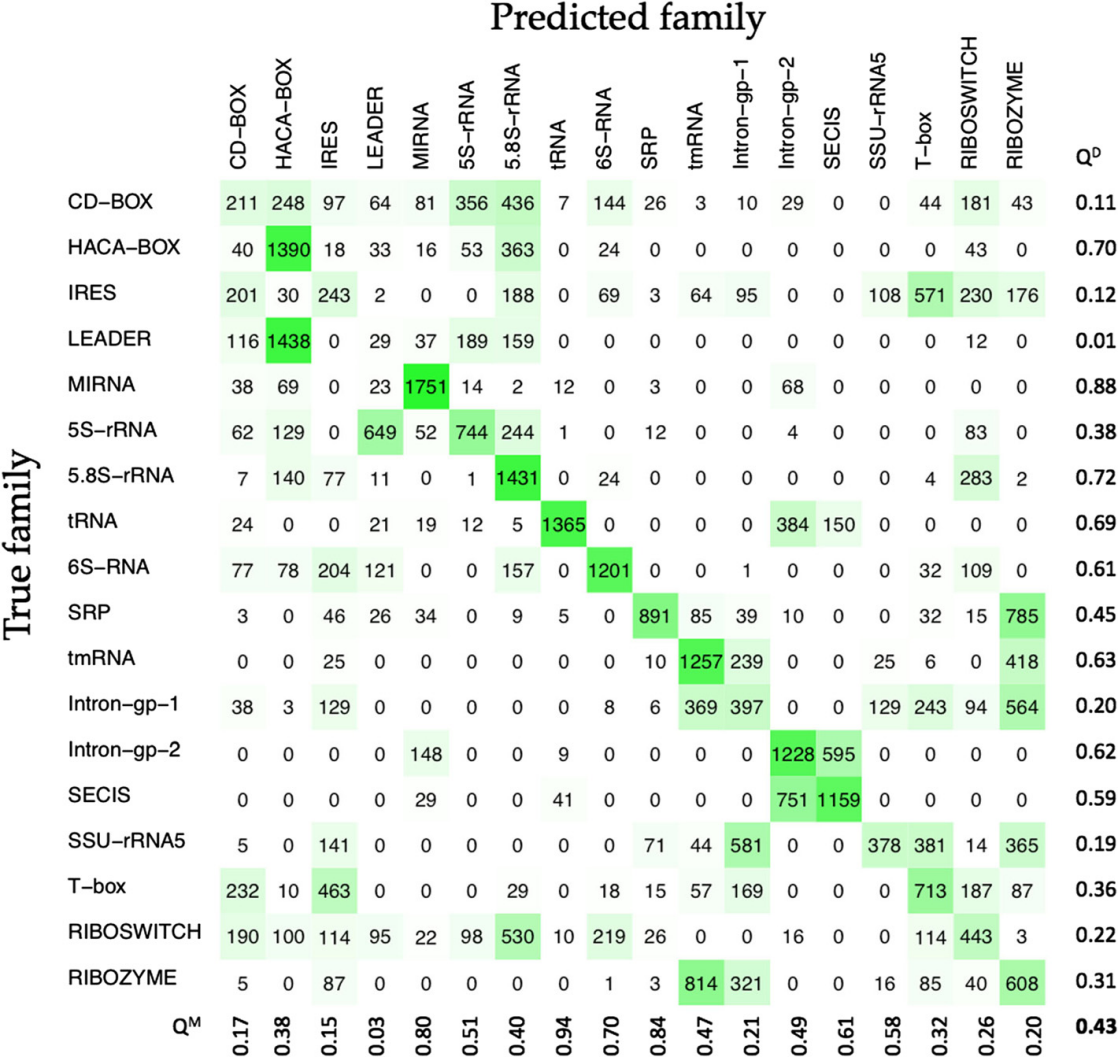
**Confusion matrix for 18 different classes of non-coding RNAs using RandomForest algorithm.** Q^D^ and Q^M^ values are showing sensitivity and specificity for each ncRNA class respectively. White to green color showing number of entries from the range of 0 to 1980.

In order to undertake a one-to-one comparison with the GraPPLE method, we created the same confusion matrix (Figure 4), as was shown in Childs *et al* [see Table 2, [30]]. Following Childs et al [30], 99 random testing sets (1980 total test sequences for each class), were incorporated into the confusion matrix (Figure 4). Comparing the performance parameters, we achieved sensitivity of 0.43 and MCC of 0.40 as compared to sensitivity of 0.33 and MCC of 0.29 achieved by the GraPPLE method. In the class-wise comparisons of all 18 classes, sensitivity of 13 classes and specificity of 14 classes of our method is higher than those of the GraPPLE method (Figure 4). It clearly shows that our RandomForest based approach performed better than the libSVM-based approach of GraPPLE method.

#### Comparison with Rfam-based covariance models (Rfam-CM)

Although, GraPPLE already compared the graph-properties based and covariance based models [30], the study employed MUSCLE based alignments, that may artificially handicap the performance of covariance models. Therefore, we used the original Rfam-based covariance models (Rfam-CM) and compared with our RNAcon method. All the sequences of different ncRNA classes used for the development of RNAcon were retrieved from Rfam (release 9.0 only). We evaluate Rfam-CM version and RNAcon on novel sequences those that were not included in release 9.0. In order to remove biasness in prediction, we have only taken new sequences that have no similarity (BLAST e-value 0.001) with sequences in Rfam (release 9.0). In order to extract non-redundant sequences, we search sequences of different classes/families in Rfam (release 11.0) against the same families in Rfam (release 9.0). Our final dataset contained sequences of different classes in new Rfam (release 11.0) that shows no similarity at BLAST e-value 0.001 with sequences in Rfam (release 9.0).

Surprisingly, Rfam-CM (release 9.0) performed unsatisfactorily on these (novel as well as non-homologous) sequences and classified only 5.35% ncRNAs correctly. When we employed RNAcon for predicting the classes of these sequences, the prediction accuracy was 25.8% (Additional file 1: Table S9). It is noteworthy that RNAcon was able to accurately predict two non-coding RNA families (HACA-BOX and miRNA), whose sequences were novel in the comparative analysis. Above analysis indicates that RNAcon can also classify non-redundant non-coding sequences, where Rfam fails to classify the same. Overall RNAcon is a useful tool, which can classify even sequences which have low sequence similarity; it will complement existing tools like Rfam-CM.

## Discussion

**Figure 5:**
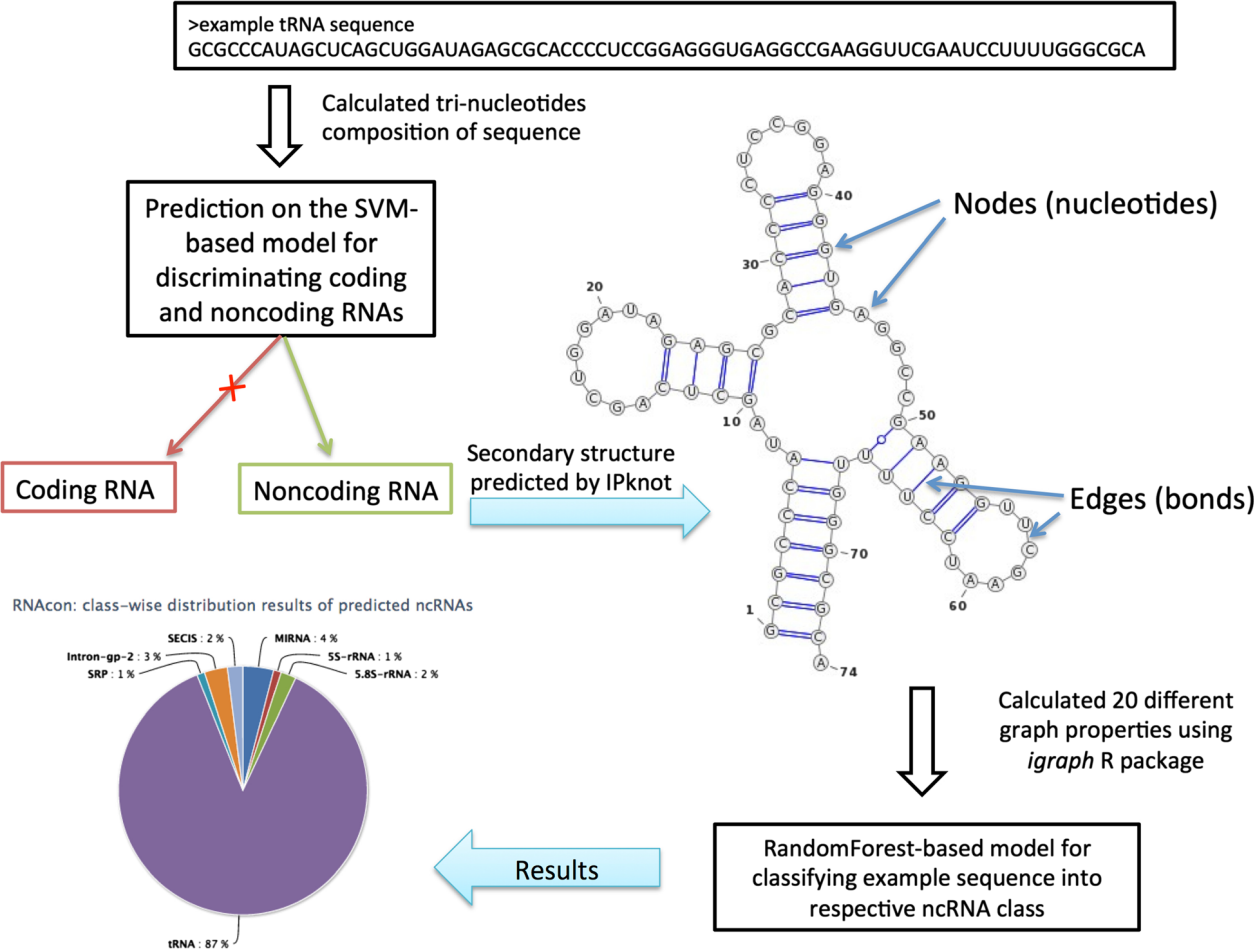
An overview of the RNAcon with an example sequence.

Knowing that biologically important functional information is present at the sequence as well as at the structure level, we investigated both the sequence-based feature set as well as the structure based graph properties to discriminate between coding and non-coding RNAs and to classify ncRNAs into different families. We initially try to discriminate sequences of ncRNAs from the sequences of protein-coding RNAs and subsequently we go on to explore the potential of a range of machine learning algorithms to classify the ncRNA sequences into different families. An overview of the algorithm of *RNAcon* method is given in the Figure 5.

For the purpose of discriminating between non-coding and coding RNA sequences, SVM based simple tri-nucleotide compositions (TNC) approach performed well. Although, nucleotide composition based approaches have been used previously by CONC [24], the study also involved the use of various other features such as peptide length, amino acid compositions, secondary structure content, percentage of residues exposed to solvent, sequence compositional entropy, number of homologs in a protein database and alignment entropy. Although biologically relevant, all these features incorporate un-necessary complexity to the problem of discriminating coding from non-coding RNAs. An advantage of using the TNC approach is that when developed into a web-based/standalone application, it efficiently discriminates coding and non-coding RNA, before we further classify them into different ncRNA classes. WEKA software [33] was used to select 14 most contributing tri-nucleotides and observed that CUA, GGG, GUA, UAA, UAC and UAG are preferred in non-coding RNAs whereas ACG, CCG, CGA, CGC, CGG, CGU, GCG and UCG are preferred in coding RNAs (Figure 1). Obviously, TNC of the stop codon UAG and UAA are more abundant in ncRNAs whereas CG containing tri-nucleotides (ACG, CCG, CGA, CGC, CGG, CGU, GCG and UCG) are more preferred in the coding RNAs.

To classify different ncRNAs, 20 different graph properties of IPknot-based predicted structures were calculated using the igraph R package. Although biological interpretation of various graph properties is not as yet established, properties related to local or global features of any ncRNA structure could reveal interesting insights of different ncRNA classes. For examples measures like betweenness and centrality could reveal the most "central" nucleotides—depicting important roles these core nodes may play in the flow of information. Global properties like degree, diameter, girth and density provide us with a holistic view of the ncRNA structures, revealing the overall "compactness" of the different classes. A thorough analysis of the biological significance if these properties could indeed prove to be beneficial. WEKA package that contains various classifiers such as BayesNet, NaiveBayes, MultilayerPerceptron, IBk, libSVM, SMO and RandomForest was used to develop and test different classification models. By applying 6 different classifiers, we found that RandomForest was the best performing classifier and achieved highest MCC of 0.40 and outperformed the MCC (0.29) of GraPPLE method [30]. The graph properties based approach performed well (Q^D^ > 0.60) for HACA-BOX, MIRNA, 5.8S-rRNA, tRNA, 6S-RNA, tmRNA and Intron-gp-1 ncRNAs while its performance was average (Q^D^ = 0.30 to 0.60) for the 5S-rRNA, SRP, T-box and RIBOZYME ncRNAs. The approach failed (Q^D^ < 0.30) to classify CD-BOX, IRES, LEADER, Intron-gp-1, SSU-rRNA5 and RIBOSWITCH (Figure 2). The reason was because most of the CD-BOX, LEADER, IRES, Intron-gp-1, SSU-rRNA5 and RIBOSWITCH sequences were wrongly predicted as 5.8S-rRNA, HACA-BOX, T-box, RIBOZYME, Intron-gp-1 and 5.8S-rRNA respectively. Many factors, such as accuracy of predicted structures and conversion of structures into the informative graph properties influence the prediction performance.

The prediction performance based comparison of *RNAcon* with three gene-calling programs indicates that RNAcon performs better in discriminating non-coding and coding sequences. Similarly, Rfam-based covariance models performed poor to classify the novel/non-similar sequences whereas comparatively structural information based graph-properties of RNAcon method performed well because graph-properties based features provide both local as well as global structural features of a particular class. The performance of Rfam-based covariance models was poor because we evaluated performance on the Rfam 11.0 sequences, which have very low similarity (Cutoff threshold 0.001 E-value) with the Rfam 9.0 database. If we evaluate all the sequences in the Rfam 11.0, which are not included in the Rfam 9.0 (non-intersecting and only present in Rfam 11.0), then performance will be much higher. Additionally, a simple algorithm for discriminating coding and noncoding RNAs is efficient enough to process thousands of RNAs in few minutes. Currently, *RNAcon* method was not developed for some newly emerging noncoding classes such as lncRNAs and CRISPR. In the future, we hope that prediction performance will be improved with more accurate and efficient structure prediction algorithms, more biologically relevant graph properties and classifiers and will also integrate the new ncRNA classes.

## Conclusion

In this study, a systematic attempt has been made to predict and classify ncRNAs. SVM based TNC approach discriminated noncoding and coding RNAs efficiently. Furthermore, graph properties based approach classifies different ncRNA classes using RandomForest classifier. Analysis showed that length of RNAs has a negative correlation with the prediction sensitivity for classifying noncoding RNAs. Comparatively, RNAcon performed well than other gene-calling programs and Rfam-based covariance models. All these prediction models have been implemented in the form of a freely available ‘*RNAcon*’ web-server/standalone.

## Methods

### Datasets

In this study, we used two different datasets for the prediction and classification of ncRNAs.

#### Dataset for the prediction of ncRNAs

We used three datasets for the development of noncoding RNA predictions- (i) main dataset, (ii) independent dataset and (iii) CONC datasets. We utilized a total of 444417 non-coding RNA sequences (all the available sequences) from Rfam release 10.0 database [38] and 97836 coding RNA sequences from RefSeq database [39]. In order to retrieve RefSeq sequences using Entrez query, we used nucleotide section of NCBI browser ( http://www.ncbi.nlm.nih.gov/nuccore) with a command (srcdb_refseq_reviewed [prop] & mRNA). It retrieved all the reviewed mRNA sequences present in the RefSeq database. Non-redundant datasets- 25% using BLASTCLUST software were created thereafter. This dataset of 40906 non-coding and 62473 coding RNA sequences was used as the main datasets. Randomly 20453 non-coding (50% of total non-coding) and 31237 coding (50% of total coding) RNA sequences were used as an independent dataset. The SVM based model training was done on the remaining 50% of both noncoding and coding RNA and performances were tested on the independent datasets. The training datasets are 25% non-redundant than testing or independent dataset. All the sequences of training dataset are less than 25% similar than any sequence of independent dataset. We also used the noncoding and coding RNA sequences from the CONC dataset [24]. This dataset was also used by the CPC method [25]. In all the prediction methods, non-coding and coding RNA sequences were used as positive and negative sets respectively.

#### Dataset for the classification of different ncRNAs

In the classification of different non-coding RNA classes, we used the previously developed dataset of GraPPLE method [30], which was originally obtained from Rfam release 9.0. This dataset contained 20% non-redundant sequences of 18 different non-coding RNA classes (CD-BOX, HACA-BOX, IRES, LEADER, MIRNA, 5S-rRNA, 5.8S-rRNA, tRNA, 6S-RNA, SRP, tmRNA, Intron-gp-1, Intron-gp-2, SECIS, SSU-rRNA5, T-box, RIBOSWITCH and RIBOZYME). Different datasets for the training and testing of each ncRNA class were used and sequence similarity between training and testing datasets was ≤ 20%.

### Nucleotide compositions

Previously, it has been shown that the composition-based approaches are useful to develop machine learning based prediction of biological sequences [24]. Most of machine learning algorithm requires fixed length of input features. Thus, we calculated mono-, di-, tri-, tetra- and penta-nucleotde compositions of 4, 16, 64, 256, and 1024 input features respectively. The major challenge is to develop efficient prediction tool with less possible input features so it is not advisable to use tetra and penta-nucleotide compositions for the predictions.

### Hybrid approach

We applied a hybrid approach for the discrimination between noncoding and coding RNAs. In this approach we used five predicted SVM scores of all approaches (MNC, DNC, TNC, TTNC and PNC) as input features and developed a separate SVM-based prediction model.

### IPknot software

We predicted the secondary structures of non-coding RNA using IPknot software. It predicts pseudoknots based on the maximizing expected accuracy [31] and the output is generated in the dot-parenthesis format of five secondary structures: open small bracket, close small bracket, open square bracket, close square bracket and dot. The small brackets, square brackets and dots denote the allowed base pair, pseudoknots and unpaired bases respectively.

### *igraph* R package and graph properties

The predicted ncRNA structures were used for the calculation of graph properties using igraph R package [34]. A total of 20 different graph properties: Articulation points, Average path length, Average node betweenness, Variance of node betweenness, Average edge betweenness, Variance of edge betweenness, Average co-citation coupling, Average bibliographic coupling, Average closeness centrality, Variance of closeness centrality, Average Burt's constraint, Variance of Burt's constraint, Average degree, Diameter, Girth, Average coreness, Variance of coreness, Maximum coreness, Graph density and Transitivity were calculated. These are the same graph properties, which were used by GraPPLE method [30] and details of all graph properties given in the Additional file 1: Table S10. The numerical values of these graph properties were used as input features for machine learning algorithms and further prediction tool development for classification of different ncRNA classes.

### Support vector machines (SVM)

In this study, we used a well-known machine learning technique *Support Vector Machine* (SVM), which is based on the structural minimization principle of statistics theory. This is supervised learning method and can be use for both classification and regression requirements [40]. It provides a number of parameters and kernels for the proper optimization of model training. The SVM^light^ Version 6.02 package [41] of SVM was used and three different (linear, polynomial and radial basis function) kernels were applied for model building. We optimized different parameters & kernels and developed efficient prediction models for the discrimination between coding and non-coding RNAs.

### WEKA package

WEKA is a single platform of various machine-learning algorithms [33]. We used WEKA 3.6.4 version, which contains different classifiers such as BayeNet, NaiveBayes, MultilayerPerceptron, IBk, libSVM, SMO and RandomForest. We applied all these classifiers for the classification of different ncRNA classes.

### Ten-fold cross-validation and performance evaluation

In the discrimination between noncoding and coding RNA, we initially used 10-fold cross validation technique. Firstly, both positive and negative samples were divided into ten subsets separately. Secondly, ten sets were created, where each set containing one positive and one negative subset. In the model learning, nine sets have been used for training and the remaining tenth set was used for testing. This step was repeated ten times and each set was used once for testing. Finally, average performance of all ten testing sets was calculated. The performances were calculated in terms of sensitivity (SN; Equation 1), specificity (SP; Equation 2), accuracy (ACC; Equation 3) and MCC (Equation 4), which are well known and have been applied earlier in various prediction methods [32,42].

(1) $$\text{Sensitivity}=\frac{\text{TP}}{\text{TP}+\text{FN}}\times 100$$

(2) $$\text{Specificity}=\frac{\text{TN}}{\text{TN}+\text{FP}}\times 100$$

(3) $$\text{Accuracy}=\frac{\text{TP}+\text{TN}}{\text{TP}+\text{FP}+\text{TN}+\text{FN}}\times 100$$

(4) $$\text{MCC}=\frac{\left(\text{TP}\right)\left(\text{TN}\right)-\left(\text{FP}\right)\left(\text{FN}\right)}{\sqrt{\left[\text{TP}+\text{FP}\right]\left[\text{TP}+\text{FN}\right]\left[\text{TN}+\text{FP}\right]\left[\text{TN}+\text{FN}\right]}}$$

Where TP, TN, FP and FN are True Positives, True Negative, False Positives and False Negatives respectively.

For the development of the classification model and its performance evaluation we followed the same procedure as was used in the GraPPLE method [30]. We took 50 random sequences from each class of ncRNA for training and thereafter developed models based on the 10-fold cross validation. Further performance of 20 test sequences from the each class was also tested. This procedure was repeated 100 times and calculated the overall average performance. The performances were calculated in terms of sensitivity (Q^D^; Equation 5), specificity (Q^M^; Equation 6) and MCC (Equation 4).

(5) $$Q_{i}^{D}=\frac{Z_{\mathrm{ii}}}{\underset{j}{\sum }Z_{\text{ij}}}$$

(6) $$Q_{j}^{M}=\frac{Z_{\text{jj}}}{\underset{j}{\sum }Z_{\text{ij}}}$$

Where Z_ij_ is an entry in a confusion matrix of 18 ncRNA classes, *i* and *j* are index for the actual and predicted family respectively.

### *RNAcon* web-server and standalone

We implemented TNC features based SVM model (parameter: -z c -t 2 -g 0.01 -c 6 -j 2) for discriminating noncoding and coding RNAs and graph properties based RandomForest model (parameter: -I 100 -K 0 -S 10) for the classification of ncRNAs into a webserver called *RNAcon*. The *RNAcon* web-server and standalone (Linux-based command-line mode) both are freely available for the help of global scientific community and available from http://crdd.osdd.net/raghava/rnacon/%20web-address.

## Competing interests

The authors declare that they have no competing interests.

## Authors’ contributions

AA calculated graph properties of noncoding RNAs. BP optimized and developed the prediction models. BP also created the backend web server and the front end user interface. GPSR conceived the project, coordinated it and refined the manuscript drafted by BP and AA. All authors have read and approved the final manuscript.

## Supplementary Material

Additional file 1: Figure S1 Average percent mono-nucleotide and di-nucleotide compositions of non-coding and coding-RNAs. **Figure S2.** Comparative average percent mono-nucleotides compositions (MNC) of different non-coding classes. **Figure S3.** Comparative average percent di-nucleotides compositions (DNC) of different non-coding RNA classes. **Figure S4.** Comparative average percent tri-nucleotides compositions (TNC) of different non-coding RNA classes for the 20% non-redundant dataset. **Figure S5.** Confusion matrix for 18 different classes of non-coding RNAs using RandomForest algorithm. **Figure S6.** Confusion matrix for 18 different classes of non-coding RNAs using MultilayerPerceptron algorithm. **Figure S7.** Confusion matrix for 18 different classes of non-coding RNAs using SMO (RBF kernel) algorithm. **Table S1.** SVM-based prediction performances (at all threshold levels) of mono-nucleotide composition (MNC) approach for the discrimination between non-coding and coding RNAs. **Table S2.** SVM-based prediction performances (at all threshold levels) of di-nucleotide composition (DNC) approach for the discrimination between non-coding and coding-RNAs. **Table S3.** SVM-based prediction performances (at all threshold levels) of tri-nucleotide composition (TNC) approach for the discrimination between non-coding and coding-RNAs. **Table S4.** SVM-based prediction performances (at all threshold levels) of tetra-nucleotide composition (TTNC) approach for the discrimination between non-coding and coding-RNAs. **Table S5.** SVM-based prediction performances (at all threshold levels) of penta-nucleotide composition (PNC) approach for the discrimination between non-coding and coding-RNAs. **Table S6.** SVM-based prediction performances (at all threshold levels) of Hybrid approach for the discrimination between non-coding and coding-RNAs. **Table S7.** Average length and prediction performance (sensitivity) of different ncRNA classes. **Table S8.** Performance of different gene-calling programs and RNAcon on the CONC dataset. **Table S9.** Comparison of Rfam-based covariance models with RNAcon using non-similar sequences between Rfam 9.0 and 11.0 release. **Table S10.** Description of the different graph properties and values of the graph properties of predicted RNA secondary structure of an example sequence (As shown in the Figure 5). Click here for file
