# Supplementary material for: Prediction and classification of ncRNAs using structural information
Source: BMC Genomics. 2014 Feb 13;15:127. doi: 10.1186/1471-2164-15-127 (PMC3925371; doi:10.1186/1471-2164-15-127)
Supplement: Additional file 1: Figure S1 — Average percent mono-nucleotide and di-nucleotide compositions of non-coding and coding-RNAs. Figure S2. Comparative average percent mono-nucleotides compositions (MNC) of different non-coding classes. Figure S3. Comparative average percent di-nucleotides compositions (DNC) of different non-coding RNA classes. Figure S4. Comparative average percent tri-nucleotides compositions (TNC) of different non-coding RNA classes for the 20% non-redundant dataset. Figure S5. Confusion matrix for 18 different classes of non-coding RNAs using RandomForest algorithm. Figure S6. Confusion matrix for 18 different classes of non-coding RNAs using MultilayerPerceptron algorithm. Figure S7. Confusion matrix for 18 different classes of non-coding RNAs using SMO (RBF kernel) algorithm. Table S1. SVM-based prediction performances (at all threshold levels) of mono-nucleotide composition (MNC) approach for the discrimination between non-coding and coding RNAs. Table S2. SVM-based prediction performances (at all threshold levels) of di-nucleotide composition (DNC) approach for the discrimination between non-coding and coding-RNAs. Table S3. SVM-based prediction performances (at all threshold levels) of tri-nucleotide composition (TNC) approach for the discrimination between non-coding and coding-RNAs. Table S4. SVM-based prediction performances (at all threshold levels) of tetra-nucleotide composition (TTNC) approach for the discrimination between non-coding and coding-RNAs. Table S5. SVM-based prediction performances (at all threshold levels) of penta-nucleotide composition (PNC) approach for the discrimination between non-coding and coding-RNAs. Table S6. SVM-based prediction performances (at all threshold levels) of Hybrid approach for the discrimination between non-coding and coding-RNAs. Table S7. Average length and prediction performance (sensitivity) of different ncRNA classes. Table S8. Performance of different gene-calling programs and RNAcon on the CONC dataset. Table S9. Compariso [file 1471-2164-15-127-S1.docx]

**Additional File 1**

**Supplementary Figures**

**Supplementary Figure S1: Average percent mono-nucleotide and di-nucleotide compositions of non-coding and coding RNAs.** The y-axis represents the log2 ratio of non-coding to coding MNC/DNC values. The height and color of the bars represents the intensity of the Log2 ratios. The greater MNC/DNC of noncoding RNAs can be visualized by the upper (red) shaded bars while the lower panel (green) shows the greater occurrence of a Mono/Di-nucleotide in coding RNA sequences.

**
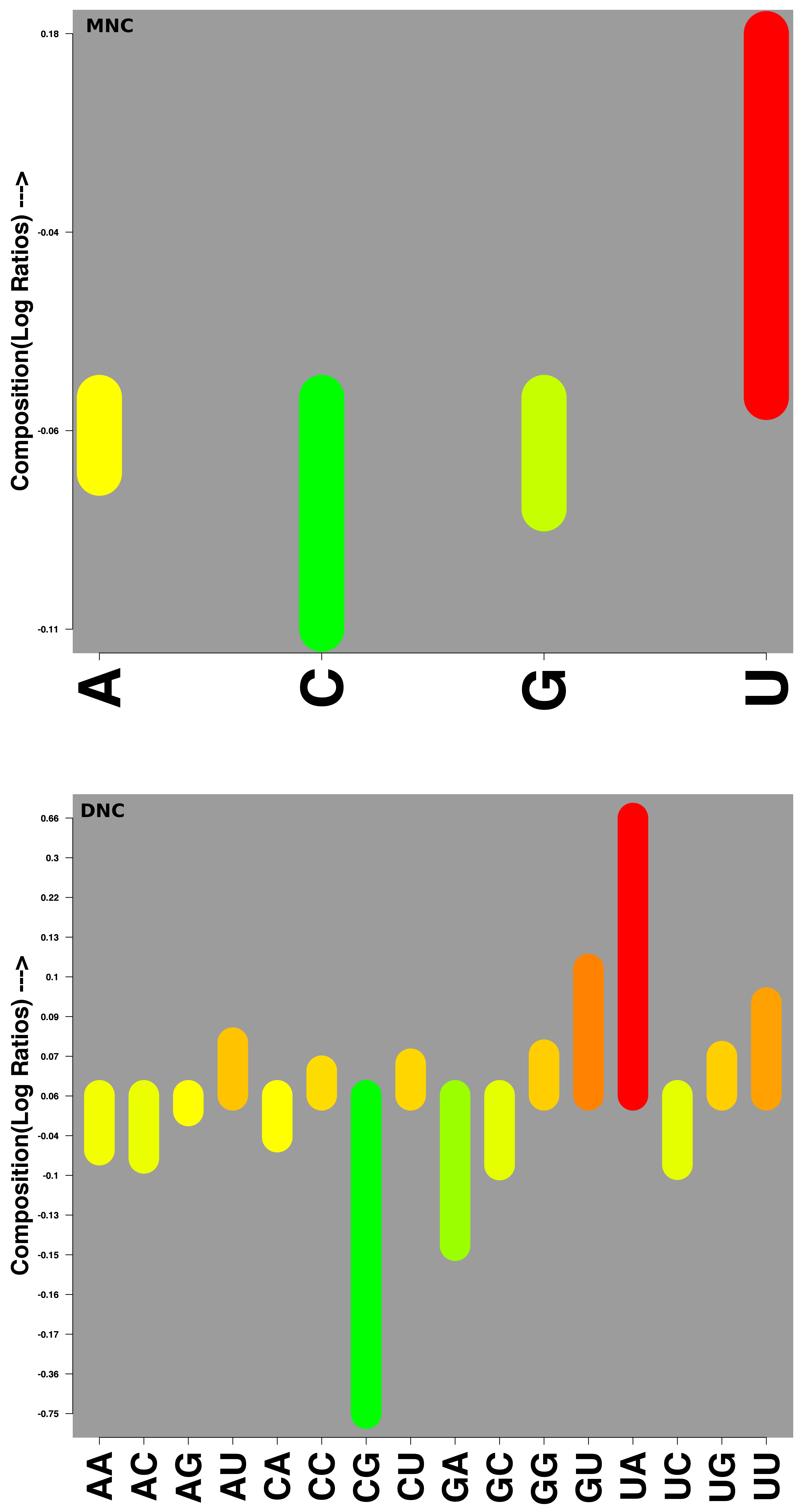
**

**Supplementary Figure S2: Comparative average percent mono-nucleotides compositions (MNC) of different non-coding RNA classes.** The MNC values can be seen for the whole dataset (**A**) and for the 20% non-redundant dataset (**B**). The diameter of the bubble is scaled according to the MNC values (the value is also numerically shown inside the bubble).

**
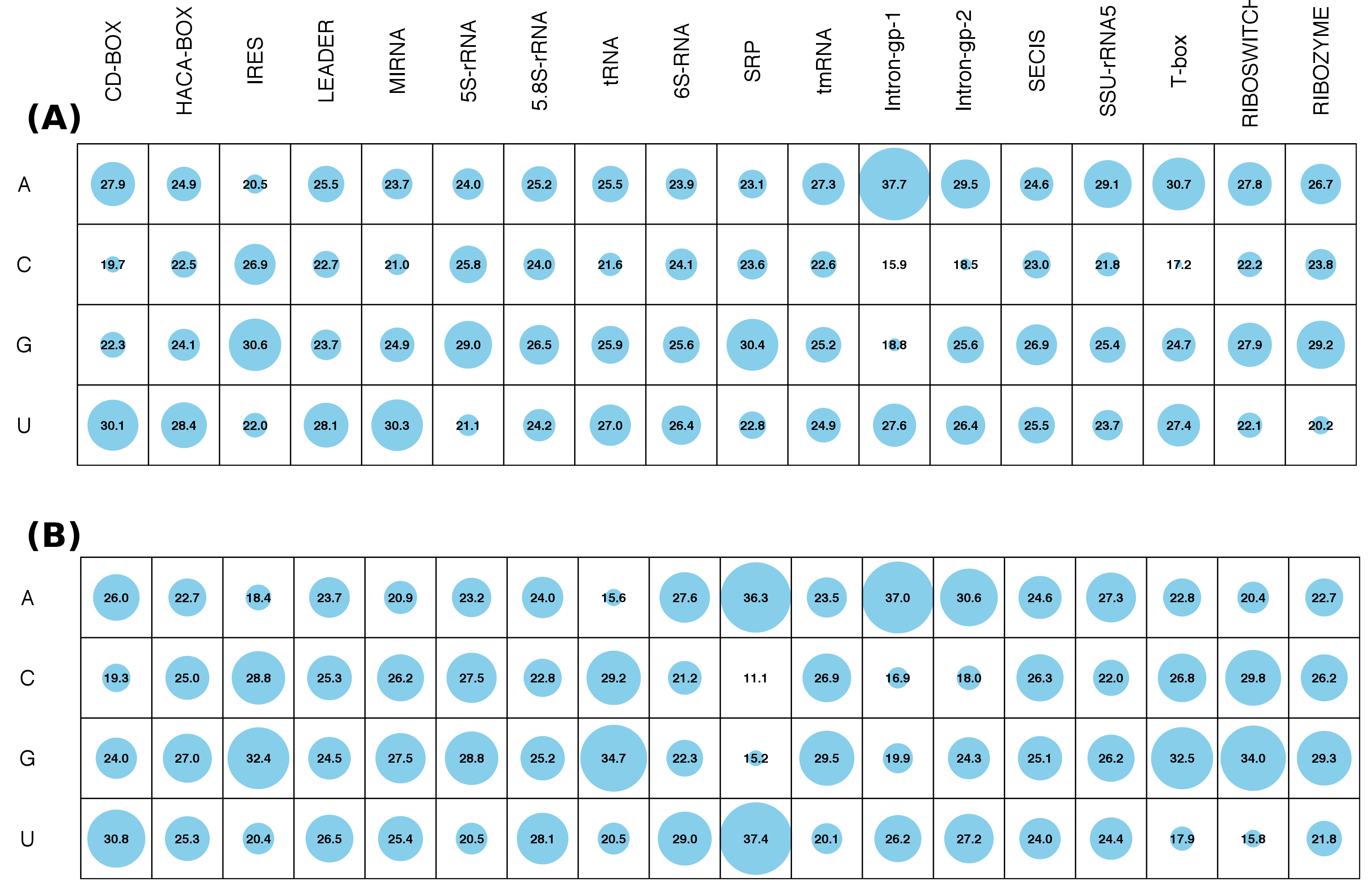
**

**Supplementary Figure S3: Comparative average percent di-nucleotides compositions (DNC) of different non-coding RNA classes.** The DNC values can be seen for the whole dataset (**A**) and for the 20% non-redundant dataset (**B**). The diameter of the bubble is scaled according to the DNC values (the value is also numerically shown inside the bubble).

**
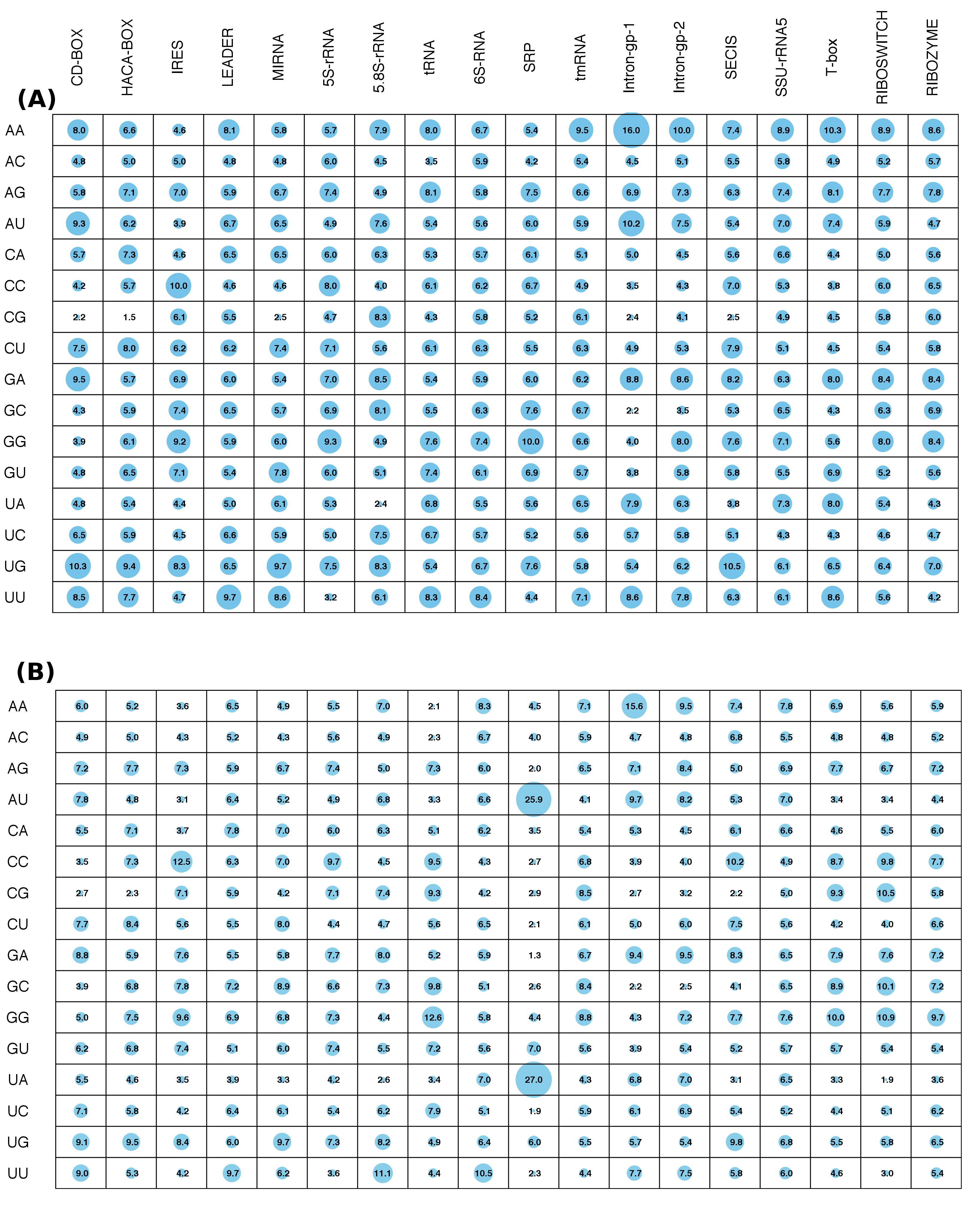
**

**Supplementary Figure S4: Comparative average percent tri-nucleotides compositions (TNC) of different non-coding RNA classes for the 20% non-redundant dataset.** The diameter of the bubble is scaled according to the TNC values (the value is also numerically shown inside the bubble).

**
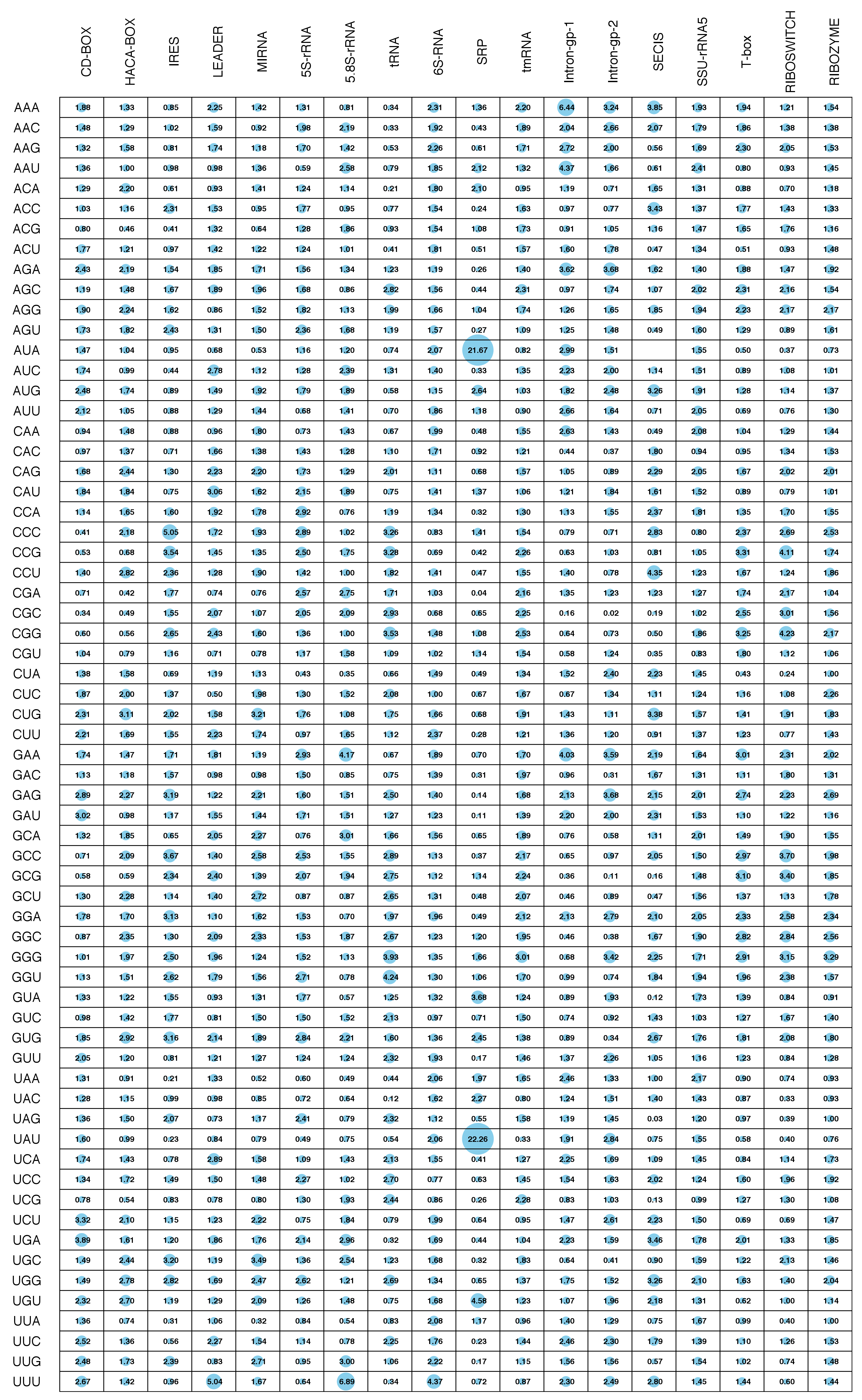
**

**Supplementary Figure S5: Confusion matrix for 18 different classes of non-coding RNAs using RandomForest algorithm.** Q^D^ and Q^M^ values are showing sensitivity and specificity for each ncRNA class respectively. White to green color showing number of entries from the range of 0 to 2000.

**
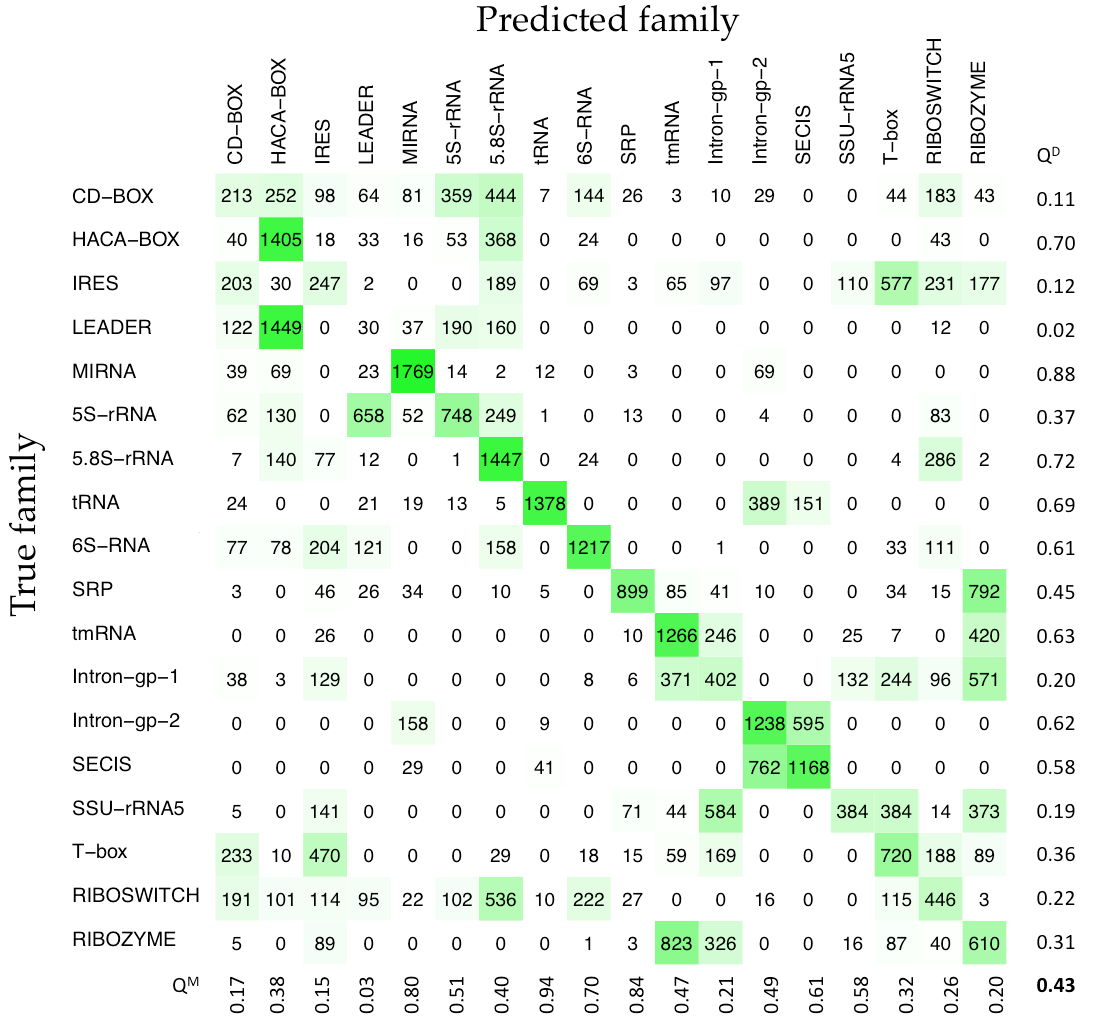
**

**Supplementary Figure S6: Confusion matrix for 18 different classes of non-coding RNAs using MultilayerPerceptron algorithm.** Q^D^ and Q^M^ values are showing sensitivity and specificity for each ncRNA class respectively. White to green color showing number of entries from the range of 0 to 2000.


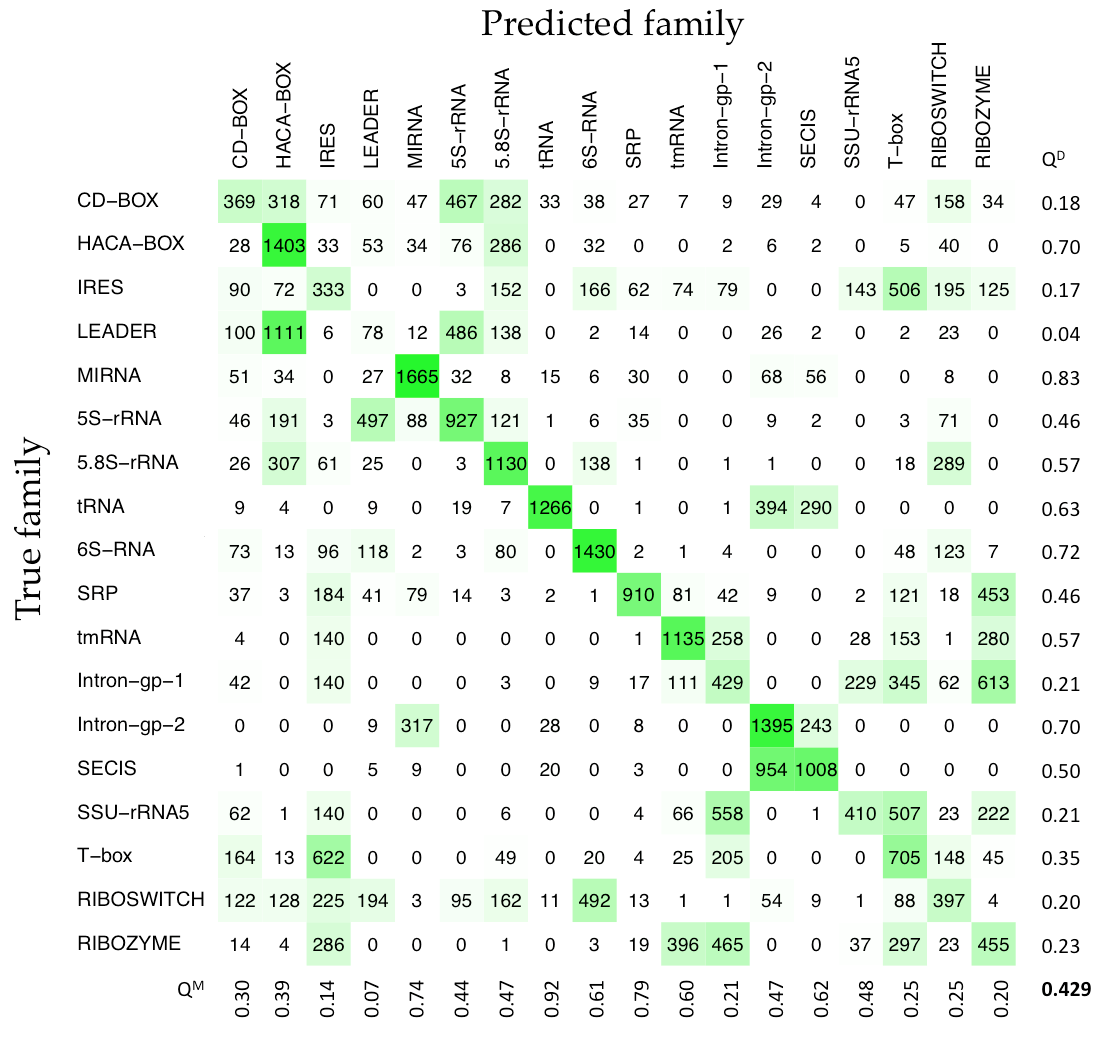


**Supplementary Figure S7: Confusion matrix for 18 different classes of non-coding RNAs using SMO (RBF kernel) algorithm.** Q^D^ and Q^M^ values are showing sensitivity and specificity for each ncRNA class respectively. White to green color showing number of entries from the range of 0 to 2000.

**
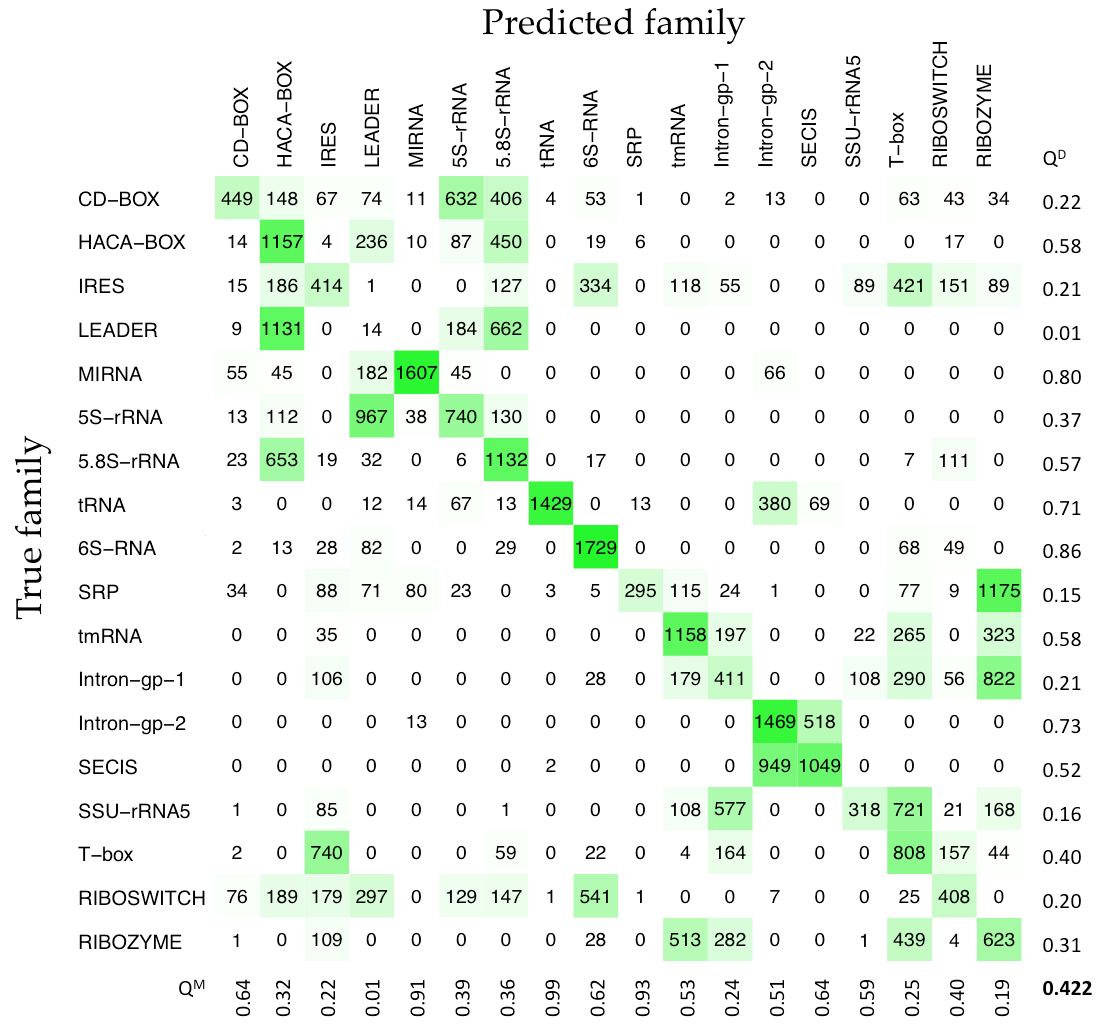
**

**Supplementary Tables**

**Supplementary Table S1: SVM-based prediction performances (at all threshold levels) of mono-nucleotide composition (MNC) approach for the discrimination between non-coding and coding RNAs.** Bold value indicates the performance with highest MCC.

| **Threshold** | **Main dataset** | | | | **Independent dataset** | | | | **CONC dataset** | | | |
| --- | --- | --- | --- | --- | --- | --- | --- | --- | --- | --- | --- | --- |
|  | **SN** | **SP** | **ACC** | **MCC** | **SN** | **SP** | **ACC** | **MCC** | **SN** | **SP** | **ACC** | **MCC** |
| **-1.0** | 83.97 | 49.44 | 63.11 | 0.34 | 84.06 | 48.52 | 62.58 | 0.33 | 73.14 | 61.79 | 65.46 | 0.33 |
| **-0.9** | 79.97 | 58.48 | 66.99 | 0.38 | 80.14 | 57.57 | 66.50 | 0.37 | 62.44 | 80.25 | 74.50 | 0.42 |
| **-0.8** | 75.95 | 66.07 | 69.98 | 0.41 | 76.07 | 65.35 | 69.59 | 0.41 | 58.95 | 83.50 | 75.58 | 0.43 |
| **-0.7** | 71.92 | 72.21 | 72.10 | 0.43 | 71.95 | 71.87 | 71.90 | 0.43 | 56.85 | 85.47 | 76.23 | 0.44 |
| **-0.6** | 67.87 | 77.25 | 73.54 | 0.45 | 67.86 | 76.96 | 73.36 | 0.45 | 54.76 | 87.08 | 76.64 | 0.44 |
| **-0.5** | 64.01 | 81.27 | 74.44 | 0.46 | **63.95** | **81.15** | **74.34** | **0.46** | 53.26 | 88.45 | 77.09 | 0.45 |
| **-0.4** | 60.22 | 84.46 | 74.87 | 0.46 | 60.08 | 84.43 | 74.80 | 0.46 | 51.54 | 89.66 | 77.36 | 0.45 |
| **-0.3** | **56.67** | **87.13** | **75.08** | **0.47** | 56.22 | 87.18 | 74.93 | 0.46 | **50.19** | **90.48** | **77.48** | **0.46** |
| **-0.2** | 53.28 | 89.32 | 75.06 | 0.47 | 52.59 | 89.42 | 74.85 | 0.46 | 48.46 | 91.29 | 77.47 | 0.45 |
| **-0.1** | 49.84 | 91.17 | 74.82 | 0.46 | 49.11 | 91.22 | 74.56 | 0.46 | 46.96 | 92.16 | 77.57 | 0.45 |
| **0.0** | 46.55 | 92.60 | 74.38 | 0.46 | 45.52 | 92.63 | 73.99 | 0.45 | 45.51 | 92.84 | 77.56 | 0.45 |
| **0.1** | 43.46 | 93.80 | 73.88 | 0.45 | 42.30 | 93.84 | 73.44 | 0.44 | 44.08 | 93.48 | 77.54 | 0.45 |
| **0.2** | 40.63 | 94.79 | 73.36 | 0.44 | 39.38 | 94.87 | 72.92 | 0.43 | 42.40 | 93.98 | 77.33 | 0.45 |
| **0.3** | 37.79 | 95.69 | 72.78 | 0.43 | 36.64 | 95.68 | 72.32 | 0.42 | 40.56 | 94.48 | 77.07 | 0.44 |
| **0.4** | 35.38 | 96.41 | 72.26 | 0.42 | 33.96 | 96.38 | 71.68 | 0.41 | 39.25 | 94.93 | 76.95 | 0.43 |
| **0.5** | 33.01 | 97.00 | 71.68 | 0.41 | 31.50 | 96.97 | 71.06 | 0.40 | 37.49 | 95.45 | 76.74 | 0.43 |
| **0.6** | 30.66 | 97.45 | 71.02 | 0.40 | 29.04 | 97.44 | 70.37 | 0.38 | 35.95 | 95.89 | 76.55 | 0.42 |
| **0.7** | 28.59 | 97.81 | 70.42 | 0.39 | 26.77 | 97.78 | 69.68 | 0.37 | 34.61 | 96.27 | 76.36 | 0.42 |
| **0.8** | 26.47 | 98.10 | 69.76 | 0.38 | 24.62 | 98.10 | 69.03 | 0.36 | 32.32 | 96.66 | 75.89 | 0.41 |
| **0.9** | 24.57 | 98.32 | 69.14 | 0.36 | 22.89 | 98.35 | 68.49 | 0.35 | 30.34 | 97.14 | 75.58 | 0.40 |
| **1.0** | 22.83 | 98.51 | 68.56 | 0.35 | 21.24 | 98.56 | 67.96 | 0.33 | 25.69 | 97.82 | 74.54 | 0.37 |

**Supplementary Table S2: SVM-based prediction performances (at all threshold levels) of di-nucleotide composition (DNC) approach for the discrimination between non-coding and coding RNAs.** Bold value indicates the performance with highest MCC.

| **Threshold** | **Main dataset** | | | | **Independent dataset** | | | | **CONC dataset** | | | |
| --- | --- | --- | --- | --- | --- | --- | --- | --- | --- | --- | --- | --- |
|  | **SN** | **SP** | **ACC** | **MCC** | **SN** | **SP** | **ACC** | **MCC** | **SN** | **SP** | **ACC** | **MCC** |
| **-1.0** | 98.97 | 88.42 | 92.60 | 0.86 | 98.95 | 87.43 | 91.99 | 0.85 | 95.36 | 66.72 | 75.96 | 0.58 |
| **-0.9** | 98.77 | 89.49 | 93.16 | 0.87 | 98.76 | 88.57 | 92.61 | 0.86 | 94.83 | 70.72 | 78.50 | 0.61 |
| **-0.8** | 98.60 | 90.45 | 93.68 | 0.88 | 98.50 | 89.78 | 93.23 | 0.87 | 93.74 | 74.06 | 80.41 | 0.64 |
| **-0.7** | 98.36 | 91.36 | 94.13 | 0.88 | 98.27 | 90.80 | 93.76 | 0.88 | 92.40 | 77.11 | 82.05 | 0.65 |
| **-0.6** | 98.14 | 92.19 | 94.54 | 0.89 | 97.99 | 91.71 | 94.19 | 0.88 | 91.09 | 79.75 | 83.41 | 0.67 |
| **-0.5** | 97.81 | 92.93 | 94.86 | 0.90 | 97.73 | 92.50 | 94.57 | 0.89 | 90.15 | 82.13 | 84.72 | 0.69 |
| **-0.4** | 97.51 | 93.65 | 95.18 | 0.90 | 97.37 | 93.22 | 94.87 | 0.90 | 88.39 | 84.32 | 85.64 | 0.70 |
| **-0.3** | **97.13** | **94.27** | **95.40** | **0.91** | 96.99 | 93.91 | 95.13 | 0.90 | 86.89 | 86.34 | 86.52 | 0.71 |
| **-0.2** | 96.69 | 94.85 | 95.58 | 0.91 | 96.51 | 94.50 | 95.29 | 0.90 | 84.98 | 88.20 | 87.16 | 0.71 |
| **-0.1** | 96.18 | 95.38 | 95.70 | 0.91 | **95.93** | **95.08** | **95.42** | **0.91** | 83.45 | 89.68 | 87.67 | 0.72 |
| **0.0** | 95.59 | 95.90 | 95.78 | 0.91 | 95.25 | 95.64 | 95.49 | 0.91 | **81.54** | **91.15** | **88.04** | **0.73** |
| **0.1** | 94.90 | 96.33 | 95.77 | 0.91 | 94.54 | 96.14 | 95.51 | 0.91 | 79.21 | 92.13 | 87.96 | 0.72 |
| **0.2** | 94.08 | 96.78 | 95.71 | 0.91 | 93.59 | 96.60 | 95.41 | 0.90 | 77.53 | 93.05 | 88.04 | 0.72 |
| **0.3** | 93.14 | 97.20 | 95.59 | 0.91 | 92.63 | 97.07 | 95.31 | 0.90 | 75.35 | 93.89 | 87.91 | 0.72 |
| **0.4** | 91.98 | 97.58 | 95.36 | 0.90 | 91.52 | 97.46 | 95.11 | 0.90 | 73.45 | 94.61 | 87.78 | 0.71 |
| **0.5** | 90.70 | 97.95 | 95.08 | 0.90 | 90.02 | 97.86 | 94.76 | 0.89 | 71.53 | 95.38 | 87.68 | 0.71 |
| **0.6** | 88.97 | 98.31 | 94.61 | 0.89 | 88.05 | 98.22 | 94.20 | 0.88 | 69.36 | 95.98 | 87.39 | 0.70 |
| **0.7** | 86.76 | 98.63 | 93.93 | 0.88 | 85.62 | 98.59 | 93.46 | 0.86 | 67.11 | 96.59 | 87.08 | 0.70 |
| **0.8** | 83.82 | 98.91 | 92.94 | 0.86 | 82.16 | 98.97 | 92.32 | 0.84 | 64.72 | 96.91 | 86.52 | 0.68 |
| **0.9** | 79.16 | 99.24 | 91.30 | 0.82 | 76.50 | 99.27 | 90.26 | 0.80 | 62.32 | 97.23 | 85.96 | 0.67 |
| **1.0** | 58.59 | 99.52 | 83.33 | 0.67 | 50.93 | 99.61 | 80.35 | 0.61 | 60.08 | 97.55 | 85.46 | 0.66 |

**Supplementary Table S3: SVM-based prediction performances (at all threshold levels) of tri-nucleotide composition (TNC) approach for the discrimination between non-coding and coding RNAs.** Bold value indicates the performance with highest MCC.

| **Threshold** | **Main dataset** | | | | **Independent dataset** | | | | **CONC dataset** | | | |
| --- | --- | --- | --- | --- | --- | --- | --- | --- | --- | --- | --- | --- |
|  | **SN** | **SP** | **ACC** | **MCC** | **SN** | **SP** | **ACC** | **MCC** | **SN** | **SP** | **ACC** | **MCC** |
| **-1.0** | 99.92 | 95.36 | 97.16 | 0.94 | 99.89 | 94.62 | 96.70 | 0.93 | 95.80 | 80.95 | 85.75 | 0.72 |
| **-0.9** | 99.89 | 96.07 | 97.58 | 0.95 | 99.87 | 95.38 | 97.16 | 0.94 | 94.98 | 83.70 | 87.34 | 0.75 |
| **-0.8** | 99.86 | 96.64 | 97.91 | 0.96 | 99.84 | 96.07 | 97.56 | 0.95 | 93.82 | 86.47 | 88.84 | 0.77 |
| **-0.7** | 99.80 | 97.14 | 98.19 | 0.96 | 99.80 | 96.61 | 97.87 | 0.96 | 92.66 | 88.79 | 90.04 | 0.79 |
| **-0.6** | 99.73 | 97.52 | 98.40 | 0.97 | 99.74 | 97.07 | 98.13 | 0.96 | 91.50 | 90.86 | 91.06 | 0.80 |
| **-0.5** | 99.66 | 97.87 | 98.58 | 0.97 | 99.64 | 97.53 | 98.37 | 0.97 | 90.41 | 92.14 | 91.58 | 0.81 |
| **-0.4** | 99.56 | 98.18 | 98.72 | 0.97 | 99.53 | 97.83 | 98.50 | 0.97 | 89.10 | 93.43 | 92.03 | 0.82 |
| **-0.3** | 99.44 | 98.45 | 98.84 | 0.98 | 99.36 | 98.15 | 98.63 | 0.97 | 87.86 | 94.43 | 92.31 | 0.82 |
| **-0.2** | 99.31 | 98.67 | 98.92 | 0.98 | 99.14 | 98.39 | 98.69 | 0.97 | **86.33** | **95.39** | **92.47** | **0.83** |
| **-0.1** | 99.13 | 98.90 | 98.99 | 0.98 | 98.91 | 98.62 | 98.73 | 0.97 | 84.64 | 96.18 | 92.46 | 0.82 |
| **0.0** | **98.90** | **99.04** | **98.98** | **0.98** | **98.65** | **98.79** | **98.74** | **0.97** | 83.22 | 96.89 | 92.48 | 0.83 |
| **0.1** | 98.60 | 99.20 | 98.97 | 0.98 | 98.34 | 98.91 | 98.68 | 0.97 | 81.68 | 97.27 | 92.24 | 0.82 |
| **0.2** | 98.27 | 99.31 | 98.90 | 0.98 | 97.90 | 99.07 | 98.61 | 0.97 | 80.04 | 97.66 | 91.97 | 0.82 |
| **0.3** | 97.91 | 99.41 | 98.82 | 0.98 | 97.48 | 99.24 | 98.55 | 0.97 | 78.09 | 98.02 | 91.59 | 0.81 |
| **0.4** | 97.47 | 99.50 | 98.70 | 0.97 | 96.90 | 99.35 | 98.38 | 0.97 | 76.33 | 98.34 | 91.24 | 0.80 |
| **0.5** | 96.87 | 99.57 | 98.50 | 0.97 | 96.25 | 99.45 | 98.19 | 0.96 | 74.53 | 98.50 | 90.76 | 0.79 |
| **0.6** | 96.15 | 99.61 | 98.24 | 0.96 | 95.46 | 99.51 | 97.91 | 0.96 | 71.69 | 98.72 | 89.99 | 0.77 |
| **0.7** | 95.39 | 99.67 | 97.98 | 0.96 | 94.66 | 99.61 | 97.65 | 0.95 | 69.03 | 98.89 | 89.25 | 0.75 |
| **0.8** | 94.56 | 99.72 | 97.68 | 0.95 | 93.60 | 99.67 | 97.26 | 0.94 | 66.44 | 99.02 | 88.50 | 0.74 |
| **0.9** | 93.55 | 99.76 | 97.30 | 0.94 | 92.46 | 99.70 | 96.83 | 0.93 | 63.45 | 99.16 | 87.63 | 0.72 |
| **1.0** | 92.38 | 99.79 | 96.86 | 0.93 | 91.09 | 99.76 | 96.33 | 0.92 | 60.00 | 99.30 | 86.62 | 0.70 |

**Supplementary Table S4: SVM-based prediction performances (at all threshold levels) of tetra-nucleotide composition (TTNC) approach for the discrimination between non-coding and coding RNAs.** Bold value indicates the performance with highest MCC.

| **Threshold** | **Main dataset** | | | | **Independent dataset** | | | | **CONC dataset** | | | |
| --- | --- | --- | --- | --- | --- | --- | --- | --- | --- | --- | --- | --- |
|  | **SN** | **SP** | **ACC** | **MCC** | **SN** | **SP** | **ACC** | **MCC** | **SN** | **SP** | **ACC** | **MCC** |
| **-1.0** | 99.97 | 96.15 | 97.66 | 0.95 | 99.95 | 95.66 | 97.36 | 0.95 | 97.19 | 77.97 | 84.17 | 0.70 |
| **-0.9** | 99.96 | 97.00 | 98.17 | 0.96 | 99.91 | 96.51 | 97.85 | 0.96 | 96.59 | 81.04 | 86.06 | 0.73 |
| **-0.8** | 99.96 | 97.64 | 98.56 | 0.97 | 99.88 | 97.07 | 98.19 | 0.96 | 96.03 | 83.79 | 87.74 | 0.76 |
| **-0.7** | 99.93 | 98.03 | 98.78 | 0.97 | 99.84 | 97.55 | 98.46 | 0.97 | 95.28 | 86.11 | 89.07 | 0.78 |
| **-0.6** | 99.90 | 98.40 | 98.99 | 0.98 | 99.81 | 97.88 | 98.65 | 0.97 | 94.53 | 88.27 | 90.29 | 0.80 |
| **-0.5** | 99.85 | 98.66 | 99.13 | 0.98 | 99.77 | 98.20 | 98.82 | 0.98 | 93.67 | 90.13 | 91.27 | 0.81 |
| **-0.4** | 99.80 | 98.91 | 99.26 | 0.98 | 99.68 | 98.49 | 98.96 | 0.98 | 92.92 | 91.70 | 92.09 | 0.83 |
| **-0.3** | 99.74 | 99.06 | 99.33 | 0.99 | 99.57 | 98.72 | 99.05 | 0.98 | 91.91 | 93.00 | 92.65 | 0.84 |
| **-0.2** | 99.62 | 99.20 | 99.36 | 0.99 | 99.46 | 98.91 | 99.13 | 0.98 | 90.60 | 94.13 | 92.99 | 0.84 |
| **-0.1** | **99.50** | **99.35** | **99.41** | **0.99** | **99.29** | **99.05** | **99.15** | **0.98** | **89.51** | **94.98** | **93.22** | **0.85** |
| **0.0** | 99.30 | 99.42 | 99.37 | 0.99 | 99.08 | 99.17 | 99.14 | 0.98 | 87.83 | 95.90 | 93.29 | 0.84 |
| **0.1** | 99.05 | 99.51 | 99.32 | 0.99 | 98.77 | 99.30 | 99.09 | 0.98 | 86.25 | 96.61 | 93.27 | 0.84 |
| **0.2** | 98.76 | 99.60 | 99.27 | 0.98 | 98.40 | 99.39 | 99.00 | 0.98 | 84.34 | 97.16 | 93.03 | 0.84 |
| **0.3** | 98.43 | 99.65 | 99.17 | 0.98 | 98.04 | 99.46 | 98.90 | 0.98 | 82.96 | 97.70 | 92.94 | 0.84 |
| **0.4** | 98.04 | 99.70 | 99.04 | 0.98 | 97.60 | 99.54 | 98.77 | 0.97 | 81.20 | 98.05 | 92.61 | 0.83 |
| **0.5** | 97.56 | 99.73 | 98.87 | 0.98 | 97.02 | 99.61 | 98.58 | 0.97 | 78.91 | 98.38 | 92.09 | 0.82 |
| **0.6** | 96.95 | 99.78 | 98.66 | 0.97 | 96.38 | 99.66 | 98.36 | 0.97 | 76.52 | 98.52 | 91.42 | 0.80 |
| **0.7** | 96.22 | 99.81 | 98.39 | 0.97 | 95.58 | 99.74 | 98.09 | 0.96 | 73.78 | 98.59 | 90.58 | 0.78 |
| **0.8** | 95.30 | 99.83 | 98.04 | 0.96 | 94.55 | 99.77 | 97.71 | 0.95 | 70.60 | 98.80 | 89.70 | 0.77 |
| **0.9** | 94.32 | 99.86 | 97.67 | 0.95 | 93.37 | 99.80 | 97.26 | 0.94 | 67.53 | 99.00 | 88.84 | 0.75 |
| **1.0** | 93.19 | 99.88 | 97.23 | 0.94 | 92.22 | 99.82 | 96.82 | 0.93 | 63.97 | 99.16 | 87.80 | 0.72 |

**Supplementary Table S5: SVM-based prediction performances (at all threshold levels) of penta-nucleotide composition (PNC) approach for the discrimination between non-coding and coding RNAs.** Bold value indicates the performance with highest MCC.

| **Threshold** | **Main dataset** | | | | **Independent dataset** | | | | **CONC dataset** | | | |
| --- | --- | --- | --- | --- | --- | --- | --- | --- | --- | --- | --- | --- |
|  | **SN** | **SP** | **ACC** | **MCC** | **SN** | **SP** | **ACC** | **MCC** | **SN** | **SP** | **ACC** | **MCC** |
| **-1.0** | 99.90 | 94.24 | 96.48 | 0.93 | 99.90 | 93.27 | 95.89 | 0.92 | 96.44 | 80.77 | 85.83 | 0.73 |
| **-0.9** | 99.88 | 95.31 | 97.12 | 0.94 | 99.87 | 94.61 | 96.69 | 0.93 | 95.88 | 84.04 | 87.86 | 0.76 |
| **-0.8** | 99.82 | 96.12 | 97.59 | 0.95 | 99.82 | 95.64 | 97.29 | 0.95 | 94.83 | 86.59 | 89.25 | 0.78 |
| **-0.7** | 99.76 | 96.73 | 97.93 | 0.96 | 99.75 | 96.34 | 97.69 | 0.95 | 93.74 | 89.29 | 90.72 | 0.80 |
| **-0.6** | 99.67 | 97.22 | 98.19 | 0.96 | 99.63 | 96.92 | 98.00 | 0.96 | 92.77 | 91.07 | 91.62 | 0.82 |
| **-0.5** | 99.55 | 97.62 | 98.39 | 0.97 | 99.44 | 97.40 | 98.20 | 0.96 | 91.80 | 92.68 | 92.40 | 0.83 |
| **-0.4** | 99.37 | 97.88 | 98.47 | 0.97 | 99.26 | 97.75 | 98.35 | 0.97 | 90.71 | 93.95 | 92.90 | 0.84 |
| **-0.3** | 99.18 | 98.15 | 98.56 | 0.97 | 99.04 | 98.06 | 98.45 | 0.97 | 89.36 | 94.86 | 93.09 | 0.84 |
| **-0.2** | 98.92 | 98.38 | 98.59 | 0.97 | **98.78** | **98.29** | **98.49** | **0.97** | **88.43** | **95.64** | **93.31** | **0.85** |
| **-0.1** | **98.62** | **98.55** | **98.58** | **0.97** | 98.43 | 98.50 | 98.47 | 0.97 | 87.00 | 96.30 | 93.30 | 0.85 |
| **0.0** | 98.22 | 98.72 | 98.52 | 0.97 | 98.07 | 98.68 | 98.44 | 0.97 | 85.69 | 96.88 | 93.27 | 0.84 |
| **0.1** | 97.82 | 98.86 | 98.45 | 0.97 | 97.60 | 98.80 | 98.33 | 0.96 | 84.61 | 97.32 | 93.22 | 0.84 |
| **0.2** | 97.36 | 98.99 | 98.34 | 0.97 | 97.07 | 98.93 | 98.20 | 0.96 | 83.00 | 97.79 | 93.01 | 0.84 |
| **0.3** | 96.84 | 99.10 | 98.20 | 0.96 | 96.30 | 99.06 | 97.97 | 0.96 | 81.20 | 98.11 | 92.65 | 0.83 |
| **0.4** | 96.22 | 99.18 | 98.01 | 0.96 | 95.74 | 99.18 | 97.82 | 0.95 | 79.21 | 98.38 | 92.19 | 0.82 |
| **0.5** | 95.62 | 99.28 | 97.83 | 0.96 | 94.85 | 99.28 | 97.53 | 0.95 | 77.15 | 98.64 | 91.70 | 0.81 |
| **0.6** | 94.82 | 99.35 | 97.56 | 0.95 | 93.88 | 99.34 | 97.18 | 0.94 | 75.09 | 98.88 | 91.20 | 0.80 |
| **0.7** | 94.00 | 99.41 | 97.27 | 0.94 | 92.90 | 99.40 | 96.83 | 0.93 | 72.25 | 99.09 | 90.42 | 0.78 |
| **0.8** | 93.04 | 99.45 | 96.92 | 0.94 | 91.81 | 99.46 | 96.43 | 0.93 | 69.40 | 99.23 | 89.60 | 0.76 |
| **0.9** | 92.09 | 99.51 | 96.58 | 0.93 | 90.47 | 99.54 | 95.95 | 0.92 | 66.37 | 99.38 | 88.72 | 0.74 |
| **1.0** | 91.01 | 99.57 | 96.18 | 0.92 | 89.08 | 99.60 | 95.43 | 0.91 | 62.77 | 99.45 | 87.61 | 0.72 |

**Supplementary Table S6: SVM-based prediction performances (at all threshold levels) of Hybrid approach for the discrimination between non-coding and coding RNAs.** Bold value indicates the performance with highest MCC.

| **Threshold** | **Main dataset** | | | | **Independent dataset** | | | | **CONC dataset** | | | |
| --- | --- | --- | --- | --- | --- | --- | --- | --- | --- | --- | --- | --- |
|  | **SN** | **SP** | **ACC** | **MCC** | **SN** | **SP** | **ACC** | **MCC** | **SN** | **SP** | **ACC** | **MCC** |
| **-1.0** | 99.88 | 98.64 | 99.13 | 0.98 | 99.92 | 97.10 | 98.22 | 0.96 | 95.17 | 88.57 | 90.70 | 0.80 |
| **-0.9** | 99.86 | 98.75 | 99.19 | 0.98 | 99.89 | 97.40 | 98.38 | 0.97 | 94.57 | 90.22 | 91.62 | 0.82 |
| **-0.8** | 99.83 | 98.86 | 99.24 | 0.98 | 99.85 | 97.66 | 98.53 | 0.97 | 93.45 | 91.77 | 92.31 | 0.83 |
| **-0.7** | 99.80 | 98.95 | 99.29 | 0.99 | 99.82 | 97.88 | 98.65 | 0.97 | 92.43 | 93.04 | 92.84 | 0.84 |
| **-0.6** | 99.78 | 99.05 | 99.34 | 0.99 | 99.79 | 98.12 | 98.79 | 0.97 | 91.39 | 93.96 | 93.13 | 0.85 |
| **-0.5** | 99.75 | 99.14 | 99.38 | 0.99 | 99.75 | 98.35 | 98.91 | 0.98 | 90.63 | 94.93 | 93.54 | 0.85 |
| **-0.4** | 99.72 | 99.20 | 99.40 | 0.99 | 99.68 | 98.50 | 98.97 | 0.98 | 89.77 | 95.62 | 93.73 | 0.86 |
| **-0.3** | 99.68 | 99.27 | 99.43 | 0.99 | 99.58 | 98.67 | 99.03 | 0.98 | **89.10** | **96.29** | **93.97** | **0.86** |
| **-0.2** | 99.62 | 99.34 | 99.45 | 0.99 | 99.51 | 98.81 | 99.09 | 0.98 | 88.20 | 96.68 | 93.94 | 0.86 |
| **-0.1** | 99.56 | 99.40 | 99.47 | 0.99 | 99.38 | 98.95 | 99.12 | 0.98 | 86.89 | 97.02 | 93.75 | 0.86 |
| **0.0** | **99.46** | **99.46** | **99.46** | **0.99** | 99.27 | 99.07 | 99.15 | 0.98 | 86.29 | 97.27 | 93.72 | 0.85 |
| **0.1** | 99.38 | 99.51 | 99.46 | 0.99 | **99.11** | **99.18** | **99.15** | **0.98** | 85.66 | 97.61 | 93.75 | 0.86 |
| **0.2** | 99.32 | 99.53 | 99.45 | 0.99 | 98.91 | 99.27 | 99.13 | 0.98 | 84.57 | 97.79 | 93.52 | 0.85 |
| **0.3** | 99.16 | 99.57 | 99.41 | 0.99 | 98.73 | 99.38 | 99.12 | 0.98 | 83.74 | 97.89 | 93.33 | 0.84 |
| **0.4** | 99.06 | 99.60 | 99.39 | 0.99 | 98.47 | 99.43 | 99.05 | 0.98 | 82.96 | 98.12 | 93.23 | 0.84 |
| **0.5** | 98.92 | 99.63 | 99.35 | 0.99 | 98.17 | 99.47 | 98.96 | 0.98 | 82.10 | 98.34 | 93.10 | 0.84 |
| **0.6** | 98.76 | 99.67 | 99.31 | 0.99 | 97.87 | 99.51 | 98.86 | 0.98 | 80.90 | 98.47 | 92.79 | 0.83 |
| **0.7** | 98.58 | 99.70 | 99.25 | 0.98 | 97.41 | 99.58 | 98.72 | 0.97 | 79.59 | 98.61 | 92.47 | 0.83 |
| **0.8** | 98.40 | 99.72 | 99.19 | 0.98 | 96.93 | 99.62 | 98.55 | 0.97 | 78.50 | 98.75 | 92.21 | 0.82 |
| **0.9** | 98.16 | 99.74 | 99.12 | 0.98 | 96.52 | 99.68 | 98.43 | 0.97 | 77.26 | 98.93 | 91.93 | 0.82 |
| **1.0** | 97.92 | 99.76 | 99.03 | 0.98 | 95.92 | 99.71 | 98.21 | 0.96 | 75.92 | 99.05 | 91.58 | 0.81 |

**Supplementary Table S7: Average length and prediction performance (sensitivity) of different ncRNA classes.**

| **Class of ncRNAs** | **Average length** | **Sensitivity** |
| --- | --- | --- |
| CD-BOX | 97.7 | 0.11 |
| HACA-BOX | 136.2 | 0.70 |
| IRES | 231.8 | 0.12 |
| LEADER | 120.8 | 0.01 |
| MIRNA | 84.6 | 0.88 |
| 5S-rRNA | 116.8 | 0.38 |
| 5.8S-rRNA | 153.4 | 0.72 |
| tRNA | 72.7 | 0.69 |
| 6S-RNA | 182.2 | 0.61 |
| SRP | 212.2 | 0.45 |
| tmRNA | 351.3 | 0.63 |
| Intron-gp-1 | 397.7 | 0.20 |
| Intron-gp-2 | 77.9 | 0.62 |
| SECIS | 64.2 | 0.59 |
| SSU-rRNA5 | 479.3 | 0.19 |
| T-box | 224.6 | 0.36 |
| RIBOSWITCH | 131.1 | 0.22 |
| RIBOZYME | 237.4 | 0.31 |

**Supplementary Table S8: Performance of different gene-calling programs and RNAcon on the CONC dataset.**

| **Performance** | **AUGUSTUS** | **GeneMark.hmm** | **Glimmer.HMM** | **RNAcon**  ***(TNC-based)*** |
| --- | --- | --- | --- | --- |
| **Non-coding RNA**  (Sensitivity; correctly predicted non-coding genes) | **99.59** | **90.22** | **95.73** | **86.25** |
| **Coding RNA** (Specificity; correctly predicted coding genes) | **19.84** | **71.16** | **71.68** | **90.52** |
| **Accuracy (%)** | **45.58** | **77.32** | **79.45** | **89.14** |
| **MCC** | **0.27** | **0.57** | **0.63** | **0.76** |

**Supplementary Table S9: Comparison of Rfam-based covariance models with RNAcon using non-similar sequences between Rfam 9.0 and 11.0 release.**

| **Non-coding RNA classes** | **Rfam 9.0** | **Rfam 11.0** | **No-hits at 0.001 E-value** | **Correctly predicted sequences** | |
| --- | --- | --- | --- | --- | --- |
|  |  |  |  | **Rfam (Covariance models)** | **RNAcon**  **(Graph properties based)** |
| **CD-BOX** | 5707 | 19503 | 4471 | 710 | 139 |
| **HACA-BOX** | 20814 | 36667 | 3786 | 132 | **1308** |
| **IRES** | 570 | 1121 | 101 | 5 | 31 |
| **LEADER** | 358 | 1388 | 188 | 33 | 38 |
| **MIRNA** | 1354 | 27624 | 24214 | 35 | **7760** |
| **5S-rRNA** | 14437 | 65908 | 627 | 208 | 300 |
| **5.8S-rRNA** | 2670 | 4687 | 36 | 21 | 33 |
| **tRNA** | 31781 | 29843 | 175 | 32 | 149 |
| **6S-RNA** | 400 | 940 | 125 | 72 | 42 |
| **SRP** | 1162 | 10097 | 25 | 4 | 2 |
| **tmRNA** | 867 | 2137 | 15 | 4 | 2 |
| **Intron Group I** | 7295 | 10424 | 4 | 4 | 2 |
| **Intron Group II** | 1464 | 4184 | 19 | 4 | 13 |
| **SECIS** | 251 | 240 | 1 | 1 | 1 |
| **SSU-rRNA5** | 24239 | 466 | 0 | 0 | 0 |
| **T-box** | 942 | 4211 | 202 | 117 | 3 |
| **RIBOSWITCH** | 5046 | 17152 | 3325 | 344 | 41 |
| **RIBOZYME** | 1901 | 11988 | 943 | 320 | 6 |
| **Total** | | | **38257** | **2046 (5.35%)** | **9870 (25.8%)** |

**Supplementary Table S10: Description of the different graph properties and values of the graph properties of predicted RNA secondary structure of an example sequence (As shown in the Figure 5).**

| **Graph property** | **Value** | **Description** |
| --- | --- | --- |
| Articulation points | 73 | A vertex in an undirected connected graph is an articulation point (or cut vertex) if removing it (and edges through it) disconnects the graph. Articulation points represent vulnerabilities in a connected network – single points whose failure would split the network into 2 or more disconnected components. |
| Average bibliographic coupling | 0.10 | The number of neighboring other vertices of two vertices (both cite). |
| Average Burt's constraint | 0.44 | It is the extent to which a node is connected to its neighbors if the neighbors are also interconnected with the nodes other neighbors. |
| Variance of Burt's constraint | 0.01 |  |
| Average closeness centrality | 0 | Shortest path between a vertex and other vertices reachable from that vertex. |
| Variance of closeness centrality | 0 |  |
| Average co-citation coupling | 0.10 | Co-citation of two vertices, if there is another vertex linking both of them. |
| Average coreness | 2.55 | The K-core of graph is a maximal sub-graph in which each vertex has at least degree k. The coreness of a vertex is k if it belongs to the k-core but not to the (k+1)-core. |
| Variance of coreness | 0.28 |  |
| Maximum coreness | 3.00 |  |
| Average degree | 3.11 | The number of edges connected to a node. |
| Average edge betweenness | 224.90 | The number of shortest paths that pass through an edge. |
| Variance of edge betweenness | 41672.87 |  |
| Average node betweenness | 313.01 | The number of shortest paths that pass through a node. |
| Variance of node betweenness | 55151.33 |  |
| Average path length | 9.58 | Number of edges between two nodes. |
| Diameter | 22.00 | Length of the longest shortest path. |
| Girth | 4.00 | If a graph forms cycles, the length of the shortest cycle. |
| Graph density | 0.04 | Ratio of number of edges and total number of possible edges in the graph. |
| Transitivity | 0 | The transitivity of a graph is based on the relative number of triangles in the graph, compared to total number of connected triplets (i.e., paths on 3 nodes) of nodes. |
